# Emotional characteristic analysis of human gait while real-time movie viewing

**DOI:** 10.3389/frai.2022.989860

**Published:** 2022-10-14

**Authors:** Nitchan Jianwattanapaisarn, Kaoru Sumi, Akira Utsumi, Nirattaya Khamsemanan, Cholwich Nattee

**Affiliations:** ^1^School of Systems Information Science, Future University Hakodate, Hokkaido, Japan; ^2^Department of Ambient Intelligence, Interaction Science Laboratories, Deep Interaction Laboratory Group, Advanced Telecommunications Research Institute International, Kyoto, Japan; ^3^Research Unit in Gait Analysis and Intelligent Technology (GaitTech), Sirindhorn International Institute of Technology, Thammasat University, Pathum Thani, Thailand

**Keywords:** emotion recognition, gait analysis, motion capturing, smart glasses, non-straight walking behavior, emotion induction, emotional movies, watching video while walking

## Abstract

Emotion recognition is useful in many applications such as preventing crime or improving customer satisfaction. Most of current methods are performed using facial features, which require close-up face information. Such information is difficult to capture with normal security cameras. The advantage of using gait and posture over conventional biometrics such as facial features is that gaits and postures can be obtained unobtrusively from faraway, even in a noisy environment. This study aims to investigate and analyze the relationship between human emotions and their gaits or postures. We collected a dataset made from the input of 49 participants for our experiments. Subjects were instructed to walk naturally in a circular walking path, while watching emotion-inducing videos on Microsoft HoloLens 2 smart glasses. An OptiTrack motion-capturing system was used for recording the gaits and postures of participants. The angles between body parts and walking straightness were calculated as features for comparison of body-part movements while walking under different emotions. Results of statistical analyses show that the subjects' arm swings are significantly different among emotions. And the arm swings on one side of the body could reveal subjects' emotions more obviously than those on the other side. Our results suggest that the arm movements together with information of arm side and walking straightness can reveal the subjects' current emotions while walking. That is, emotions of humans are unconsciously expressed by their arm swings, especially by the left arm, when they are walking in a non-straight walking path. We found that arm swings in happy emotion are larger than arm swings in sad emotion. To the best of our knowledge, this study is the first to perform emotion induction by showing emotion-inducing videos to the participants using smart glasses during walking instead of showing videos before walking. This induction method is expected to be more consistent and more realistic than conventional methods. Our study will be useful for implementation of emotion recognition applications in real-world scenarios, since our emotion induction method and the walking direction we used are designed to mimic the real-time emotions of humans as they walk in a non-straight walking direction.

## 1. Introduction

Recently, research on emotion recognition and analysis has gained much popularity due to its usefulness. This technology makes it possible to implement several types of applications, which include improving the quality of human-robot interaction (Yelwande and Dandavate, 2020), evaluating customer satisfaction (Bouzakraoui et al., 2019), detecting suspicious behaviors for crime prevention (Anderez et al., 2021), and assessing student engagement during online classes (Tiam-Lee and Sumi, 2019).

Due to the popularity of emotion analysis research, a specific research field called *Affective Computing* (Picard, 2000) has emerged. This research field focuses on giving computers the capability to understand human emotion as well as to generate human-like affects for various applications. Several affective computing applications have been proposed in recent years. For example, in the education field, an affective computing algorithm can be applied to a program of online exercises by analyzing students' emotions and interacting with the students so they can study more effectively while also improving their mental health (Tiam-Lee and Sumi, 2019).

In security applications, gait analysis is also useful in crime prevention. Since CCTV systems and security cameras are already standard equipment widely installed in many places, due to the advances in computer vision and machine learning technology, human gait can be analyzed quickly using on-board computation devices. Identifying suspicious behaviors can thus be carried out effectively (Anderez et al., 2021). Smart Visual Surveillance applications can be implemented using gait analysis, including re-identification and forensic analysis, since gaits can be captured at a distance without the awareness or cooperation of the subjects (Bouchrika, 2018).

Human emotion analysis is also useful for improving the experience of human-robot interaction. Nowadays, robot usage is increasing in many tasks and situations including delivery tasks, warehousing tasks, and so on. Making robots move in crowded environments without disturbing or annoying humans, through choosing appropriate paths, remains a vital issue to tackle. By integrating emotion recognition of humans with robot movement strategy, a socially aware robot can be achieved. A socially aware robot can minimize its interference with humans while improving the user's quality of life (Yelwande and Dandavate, 2020).

In the past, emotion prediction could be performed using human observers (Montepare et al., 1987). However, using human observers is time consuming and not sufficiently consistent for use in real-world applications. Automatic emotion recognition, which is a more suitable and accurate approach, has thus been developed (Stephens-Fripp et al., 2017). Most publicly available methods nowadays use facial expressions as features for emotion analysis and prediction. Emotion prediction using facial features has good accuracy in some situations, but it still has limitations (Busso et al., 2004). For example, in situations such as a noisy environment, facial features are difficult to obtain, and high-quality facial images cannot be captured with standard security cameras. In some cases, if the subject has a mustache, beard, or eyeglasses, these can interfere with emotion recognition that depends on facial expressions. Emotion intensity is another important issue, since some subjects do not express intense feelings on their faces. Due to these limitations, emotion prediction techniques based on facial features are suitable for use only in limited situations. If face images can be clearly captured, such as when the subject is facing forward near the camera, facial emotion recognition is an appropriate choice. If the face images cannot be clearly captured, other features might be better for implementation of emotion recognition in reality.

Gait and posture are known as movement patterns of the human body as people walk or perform activities. There are no requirements for high-resolution images or video. Gait and posture features can be collected without interfering with the normal life of humans. Furthermore, gait and posture data can be collected without the subjects' awareness. These features make gait and posture recognition successful in many applications such as human identification (Khamsemanan et al., 2017; Limcharoen et al., 2020), human re-identification (Limcharoen et al., 2021), age estimation (Lu and Tan, 2010; Zhang et al., 2010; Nabila et al., 2018; Gillani et al., 2020), and gender recognition (Isaac et al., 2019; Kitchat et al., 2019). Therefore, human gait and posture are appropriate features for recognition of human emotions as shown in several previous studies (Montepare et al., 1987; Janssen et al., 2008; Roether et al., 2009; Karg et al., 2010; Barliya et al., 2013; Venture et al., 2014; Li B. et al., 2016; Li S. et al., 2016; Zhang et al., 2016; Chiu et al., 2018; Quiroz et al., 2018).

The objective of this study is to analyze the differences in human gaits and postures under different emotions as subjects walk in a non-straight walking path. Several experiments were conducted to investigate the differences in body-part movement under different emotions and to verify whether these differences in movement could be used to identify the current emotion of a subject. The participants in our experiments consist of male and female undergraduate university students. They were asked to walk with their natural postures in a non-straight walking path while watching emotional videos. Conventional methods where videos are shown to the subjects before walking pose the risks of the induced emotions not being consistent and not lasting until the end of walking (Kuijsters et al., 2016). Moreover, if we show videos on a normal screen while the subject walks in a non-straight path, the subject would need to bend or turn his or her head to watch the videos on the screen. With the proposed method, the subjects can see the videos directly on HoloLens 2, so they can walk in a natural way and watch the video at the same time. In addition, the induced emotion will be more consistent and last until the end of walking. In this study, gait data were analyzed using one-way and multi-factor Analysis of Variance (ANOVA) as well as Linear Regression Analysis.

Experiments and analyses in this study were conducted to prove two hypotheses. The first hypothesis is that different emotions have different effects on body-part movements while subjects walk, so the emotions of subjects can be recognized from their walking postures. The second hypothesis is that the movements of the left and right body parts are not symmetric if the subjects walk in a non-straight path, causing one side of the body to reveal the current emotion of a subject better than the other side. Since our study focuses on human gait analysis while the subjects walk in a non-straight walking path, it is dissimilar to most other conventional studies that analyze human gait only when the subjects walk in a straight walking path (Janssen et al., 2008; Michalak et al., 2009; Roether et al., 2009; Karg et al., 2010; Gross et al., 2012; Barliya et al., 2013; Destephe et al., 2013; Venture et al., 2014; Li B. et al., 2016; Li S. et al., 2016; Sun et al., 2017).

In short, our experiments and analyses show that human gait and posture are different under different emotions, especially with left arm-swing movements.

This article is organized as follows. Section 2 gives an overview of other works related to ours. Section 3 explains the method, equipment and materials we used to collect data for our analysis. Section 4 shows the detailed procedure of how we preprocessed the data we collected. Section 5 describes the method we used to extract gait features. Section 6 describes the statistical methods used to analyze our gait data and the results of each method. Section 7 discusses and analyzes the results we found from statistical analyses. Finally, Section 8 summarizes everything we have accomplished in this study.

## 2. Related works

Studies on emotion recognition are very popular, and several research projects on this topic have been proposed in recent years due to its potential usefulness. Most of the proposed emotion recognition methods are based on facial expression. These techniques can achieve accurate results for specific applications. However, emotion recognition using facial features still have limitations in some real-world usages, as mentioned in Section 1. We found that fewer studies focusing on emotion recognition have used gait and posture features than facial features. In this study, related works that are useful and relevant to our study are reviewed.

Xu et al. (2020) conducted a survey to investigate many studies on gait analysis. They found that gait analysis could be used not only for identification of subjects but also for the prediction of subjects' current emotions. They found that humans walking under different emotions show different characteristics. By using this information, automatic emotion recognition can be achieved. There are several advantages to using gait compared with traditional biometrics such as facial features, speech features, and physiological features. Gait can be observed from far away without a subject's awareness. Gait is difficult to imitate. Gait can also be obtained without a subject's cooperation. Due to these advantages, gait is a very effective type of expression that can be used for automatic emotion recognition. Gait can be recorded using many types of devices. For example, a force plate can be used for recording velocity and pressure data (Janssen et al., 2008). Infrared light barrier systems also perform well in recording velocity data (Lemke et al., 2000; Janssen et al., 2008). Motion capturing systems, e.g., Vicon, can capture coordinate data accurately using markers attached to the body (Michalak et al., 2009; Roether et al., 2009; Karg et al., 2010; Gross et al., 2012; Barliya et al., 2013; Destephe et al., 2013; Venture et al., 2014). Microsoft Kinect is another efficient tool that can capture the human skeleton by processing a depth image with a color image to predict the position of body joints (Li B. et al., 2016; Li S. et al., 2016; Khamsemanan et al., 2017; Sun et al., 2017; Kitchat et al., 2019; Limcharoen et al., 2020, 2021). An accelerometer sensor on a wearable device such as a smartphone or smart watch can also record the movement data for gait analysis (Zhang et al., 2016; Chiu et al., 2018; Quiroz et al., 2018). After gait data collection, there are several preprocessing steps that can be used. For instance, a low-pass Butterworth filter (Destephe et al., 2013; Kang and Gross, 2015, 2016) or sliding window Gaussian filtering (Li B. et al., 2016; Li S. et al., 2016). Data transformation from the time domain to others such as Discrete Fourier Transform (Li B. et al., 2016; Li S. et al., 2016; Sun et al., 2017) or Discrete Wavelet Transform are also widely used (Ismail and Asfour, 1999; Nyan et al., 2006; Baratin et al., 2015). Gait features are categorized into Spatiotemporal Features, such as stride length, velocity, step width, and step length, and Kinematic Features, such as coordinate data, joint angles, and angular range of motion. Some approach involves dimension reductions of gait features such as Principal Component Analysis (Shiavi and Griffin, 1981; Wootten et al., 1990; Deluzio et al., 1997; Sadeghi et al., 1997; Olney et al., 1998). Finally, the emotion recognition phase can be performed using many popular techniques, e.g., Multilayer Perceptrons (Janssen et al., 2008), Naive Bayes (Karg et al., 2010; Li B. et al., 2016; Li S. et al., 2016), Nearest Neighbors (Karg et al., 2010; Ahmed et al., 2018), Support Vector Machine (Karg et al., 2010; Li B. et al., 2016; Li S. et al., 2016; Zhang et al., 2016; Chiu et al., 2018), and Decision Tree (Zhang et al., 2016; Ahmed et al., 2018; Chiu et al., 2018). As for the results, useful findings were derived from many of the studies they surveyed. For happiness, the subject steps faster (Montepare et al., 1987), strides are longer (Halovic and Kroos, 2018), arm movement increases (Halovic and Kroos, 2018), and joint angle amplitude increases (Roether et al., 2009). For sadness, the arm swing decreases (Montepare et al., 1987), torso and limb shapes contract (Gross et al., 2012), and joint angles are reduced in amplitude (Roether et al., 2009). Many gait analysis studies have been proposed in recent decades. Several applications can be achieved by analyzing human gait. The following examples are illustrative: human identification or re-identification (Khamsemanan et al., 2017; Limcharoen et al., 2020, 2021), gender prediction (Isaac et al., 2019; Kitchat et al., 2019), emotion prediction (Janssen et al., 2008; Xu et al., 2020), and mental illness prediction (Lemke et al., 2000; Michalak et al., 2009). Several of the above methods collected gait data by such means as a force plate, a light barrier, a motion-capturing system, a video camera, or an accelerometer. We focus only on methods that extract 3-dimensional coordinates, binary silhouette, and body part angles as gait features, since these gait features are sensitive to walking patterns. Most current studies propose using a straight walking path in their experiments to achieve high-quality gait data (Sadeghi et al., 1997; Lemke et al., 2000; Janssen et al., 2008; Michalak et al., 2009; Roether et al., 2009; Barliya et al., 2013; Venture et al., 2014; Kang and Gross, 2015, 2016; Li B. et al., 2016; Li S. et al., 2016; Sun et al., 2017; Chiu et al., 2018). However, a few studies have used a free-style walking path, where subjects can choose any walking pattern they want instead of straight walking (Khamsemanan et al., 2017; Kitchat et al., 2019; Limcharoen et al., 2020, 2021). By developing methods for free-style walking data, there are greater opportunities to implement the proposed methods in a real-world scenario, in which humans walk without awareness of being observed in public spaces. Such methods are also motivated by the difficulty of obtaining adequate straight-walking data in noisy environments compared with free-style walking data.

In our study, we show emotion-inducing videos to subjects using Microsoft HoloLens 2 smart glasses while they walk. We were also concerned whether human gait could suffer from interference due to watching videos using smart glasses. These concerns were related to studies that measured gait performance of subjects while using smart glasses in performing attention-demanding tasks while walking, and there are some findings on this that should be considered. For example, level-walking performance was not affected in comparison to using a paper-based display and baseline walking. In addition, subjects walked more conservatively and more cautiously when crossing obstacles (Kim et al., 2018). Unfortunately, adverse impacts such as walking instability can occur when using smart glasses, but the stability issue was not as significant as when using a smartphone and a paper-based system (Sedighi et al., 2018, 2020).

Based on these related studies, we decided to use Microsoft HoloLens 2 for displaying emotional videos to our participants while they walked in the recording area, despite the possibility that some adverse effects, such as walking instability, could occur when using smart glasses while walking. We coped with this issue by asking the participants to take one rehearsal walk through the walking area without wearing HoloLens 2 to make them familiar with the walking space and another rehearsal walk while wearing HoloLens 2 without displaying anything to make them familiar with walking while wearing smart glasses at the same time. For the walking pattern, straight walking should result in cleaner gait data but it has more limitations when implemented in real-world scenarios. On the other hand, walking freely without any path guidance would be difficult for the subjects. Since they have to concentrate on the video content shown by HoloLens 2 while walking, if they also need to determine the walking path at the same time, they cannot focus well on the video content and their gait can be affected by interference. Therefore, we decided to use a lax circular walking path for our experiments. By walking circularly in clockwise or counter-clockwise direction without marking the path line on the floor, we can have both straight walking and non-straight walking data in a single walking trial.

## 3. Data collection

In most previous studies in the fields of emotional recognition and analysis, participants were asked to walk in a straight line after watching emotional movies or asked to walk in a straight line while thinking about personal experiences. Various issues arise in these settings. In cases where participants were asked to walk after watching emotional videos, it is possible that some participants do not sustain the same emotions toward the end of the walk or do not have the same emotion at all after watching the videos. These conditions can lead to inaccurate relationships between gaits and emotions. In cases where participants were asked to feel certain ways using their personal experiences, it is also possible that some participants cannot recall their feelings well enough for them to be reflected in their body movements. These problems can lead to faulty information.

To eliminate the above issues leading to faulty information and inaccurate relationships between gaits and emotions, our experiments are designed so that participants are constantly exposed to emotion-inducing videos while walking. We used the latest smart glasses technology, i.e., Microsoft HoloLens 2, to show videos to subjects while they were walking. To the best of our knowledge, no currently proposed study has ever used this kind of emotion induction method. By using HoloLens 2 for viewing videos, subjects can see the room environment and the videos at the same time. Because we show emotional videos to participants while they walk, the results are closer to real-life situations when a subject sees certain events and feels a certain emotion due to those events. In other words, we attempted to simulate the real-time emotions of the participants by showing emotion-inducing videos while they were walking. Moreover, the intensity of the induced emotions should be more consistent than with previous methods that showed emotional videos to subjects prior to walking trials.

### 3.1. Equipment for data collection

Currently, there are two main types of motion-capturing equipment: marker-less and marker-based devices. Marker-less devices are more convenient to use in real-life situations because there is no need to attach any equipment to the subject's body. Coordinates of body parts are calculated by image processing technology using depth data recorded by an infrared camera together with RGB images from a color camera. For the marker-based type, several markers must be attached to the subject's body at the desired positions, such as on the head, hand, or elbow. A marker-based device is more complex to set up because it requires several cameras to capture the infrared reflection from the markers attached to the subject's body for reconstruction of the markers' coordinates in 3-dimensional space. However, the body-tracking accuracy of a marker-less device is lower than that of a marker-based type because a marker-less system predicts the position of each body part while a marker-based system uses the actual position obtained from several cameras.

**Figure 1:**
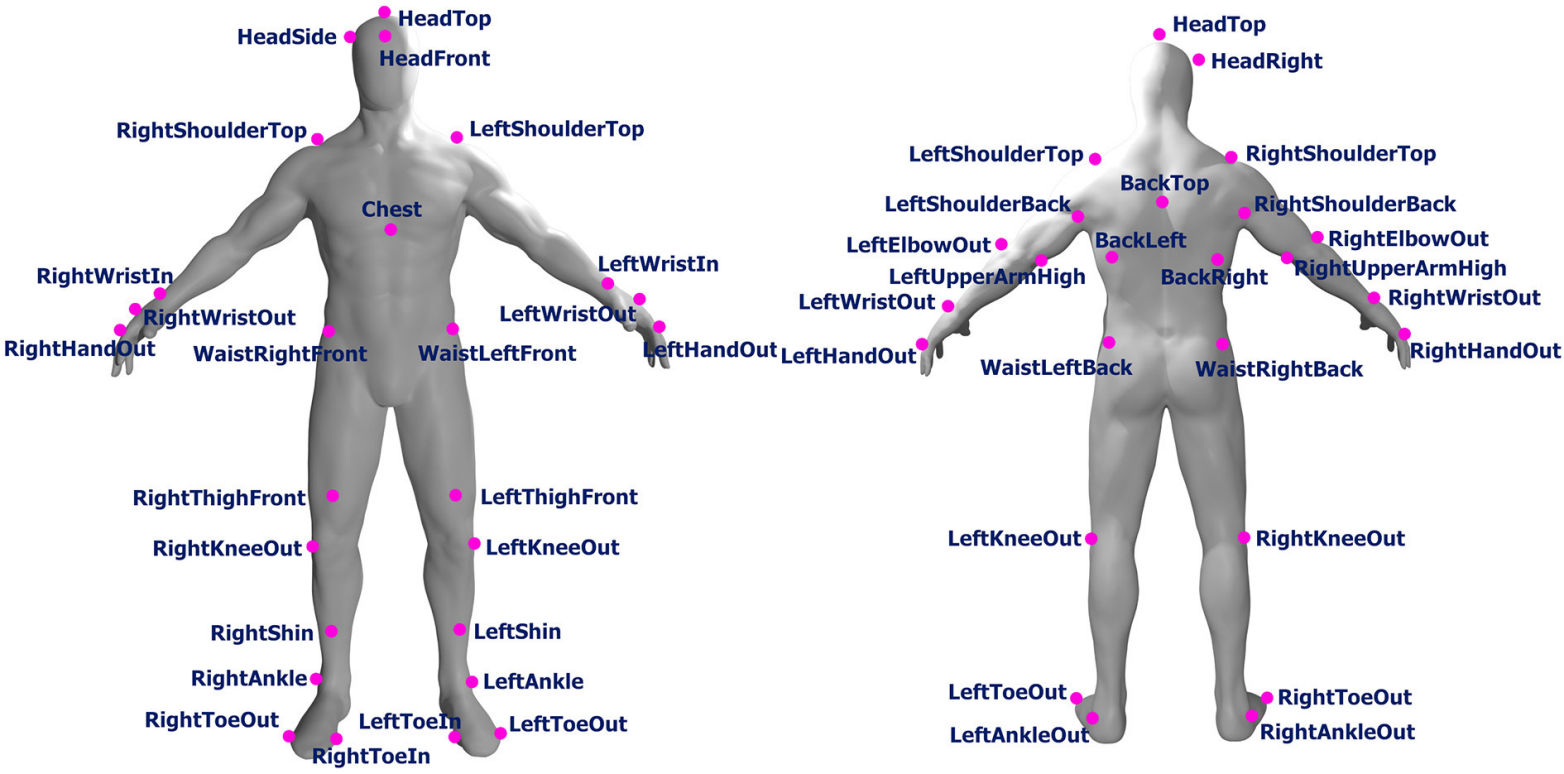
Position of front and back markers (original human figure source: dox012 on Sketchfab^1^).

**Figure 2:**
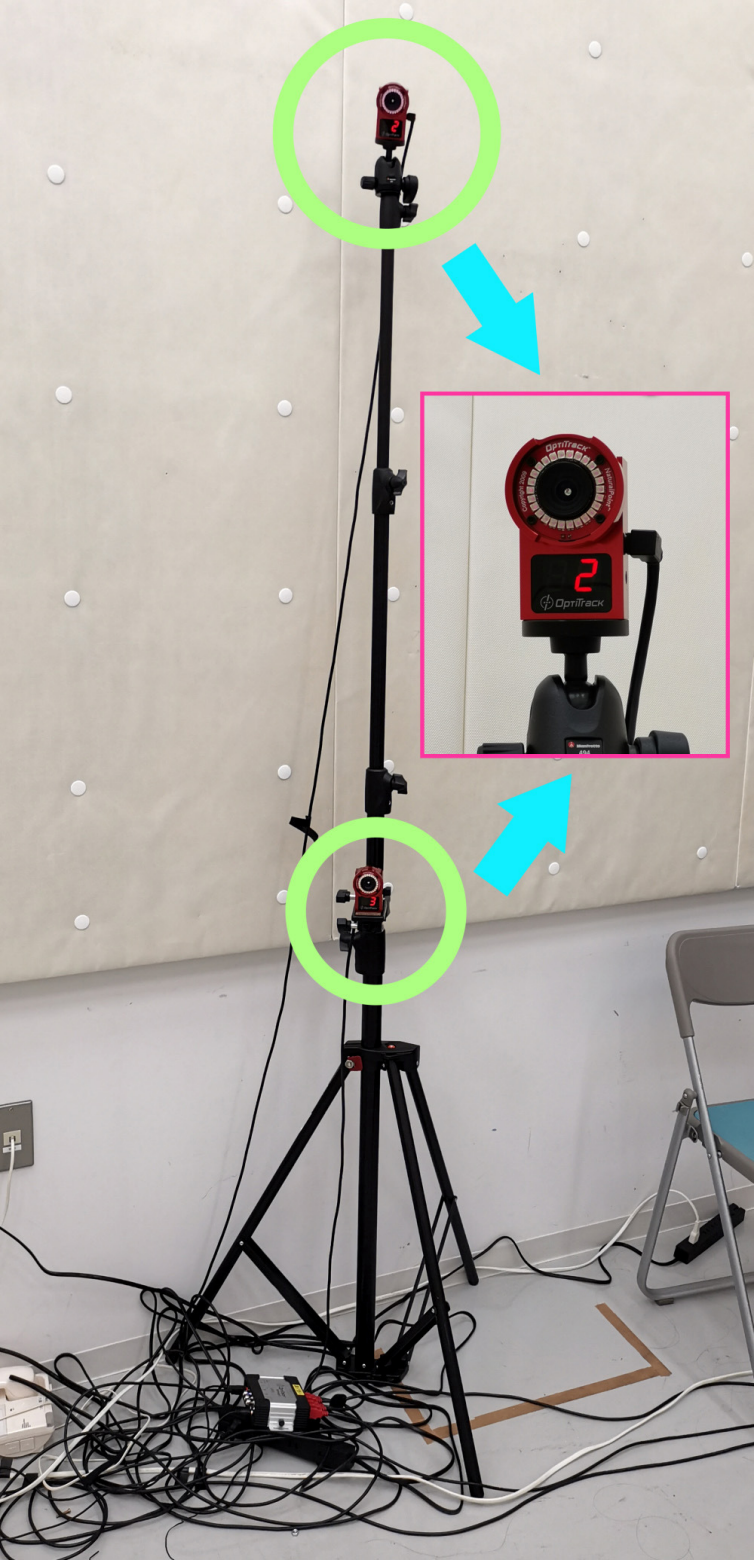
Two OptiTrack Flex 3 cameras installed on one camera stand at different heights.

In this study, we used *OptiTrack*, a well-known marker-based motion-capturing system, for our data collection. Fourteen OptiTrack Flex 3 cameras were used in our experimental design. We used the baseline marker set of 37 markers, which is the standard configuration for human skeleton tracking. With this baseline marker set configuration, the 37 markers were attached to each subject's body. The names of the markers are listed in Table 1, and the positions of the markers are shown in Figure 1. Figure 2 shows an image of how these OptiTrack cameras were installed.

**Table 1:** List of OptiTrack baseline markers.

HeadTop	
HeadFront	
HeadSide	
BackTop	
Chest	
Back	Left
	Right
WaistFront	Left
	Right
WaistBack	Left
	Right
ShoulderBack	Left
	Right
ShoulderTop	Left
	Right
ElbowOut	Left
	Right
UpperArmHigh	Left
	Right
WristOut	Left
	Right
WristIn	Left
	Right
HandOut	Left
	Right
ThightFront	Left
	Right
KneeOut	Left
	Right
Shin	Left
	Right
AnkleOut	Left
	Right
ToeOut	Left
	Right
ToeIn	Left
	Right

### 3.2. Recording environment

**Figure 3:**
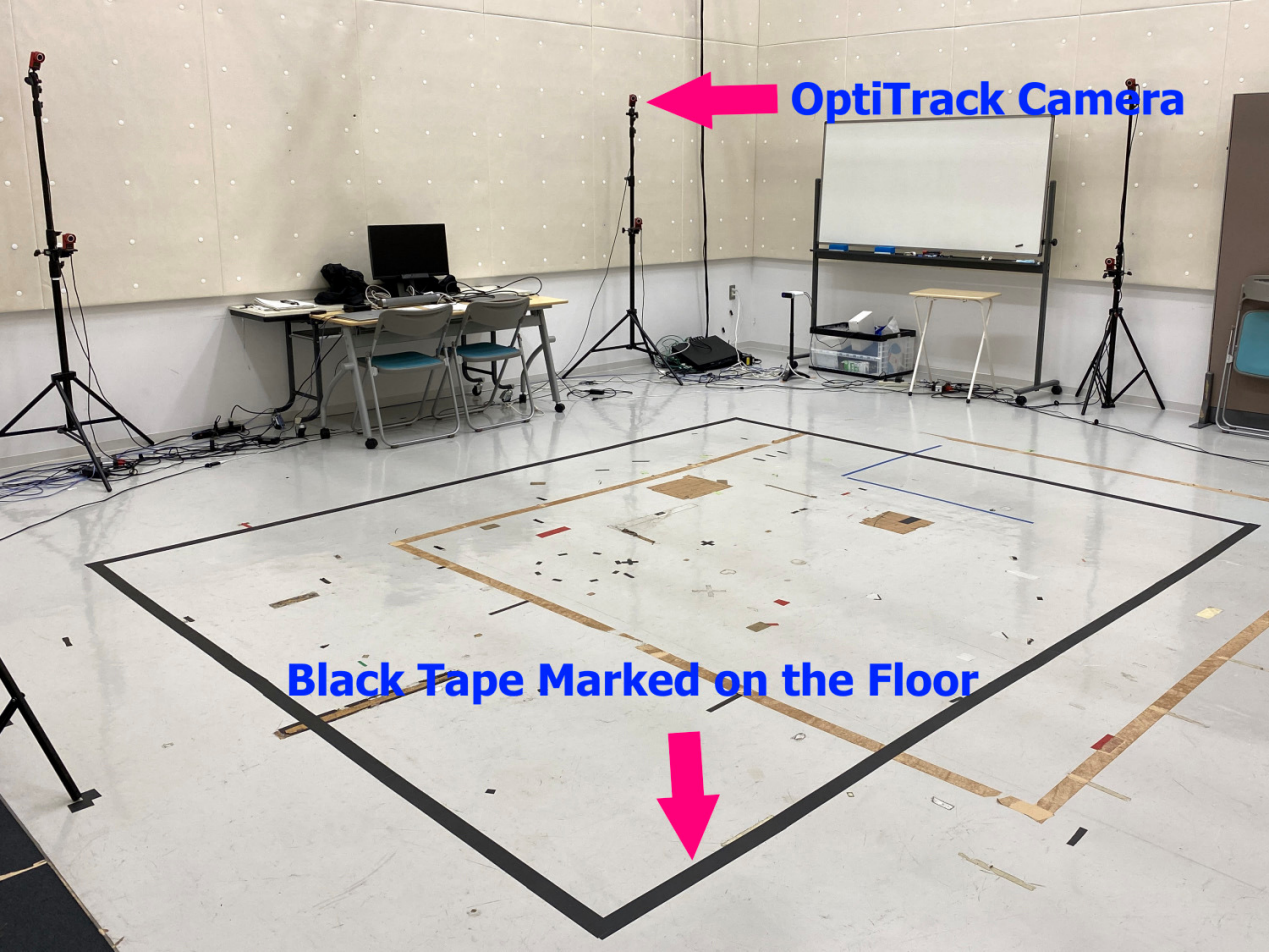
Rectangular walking area marked with black tape on the floor.

**Figure 4:**
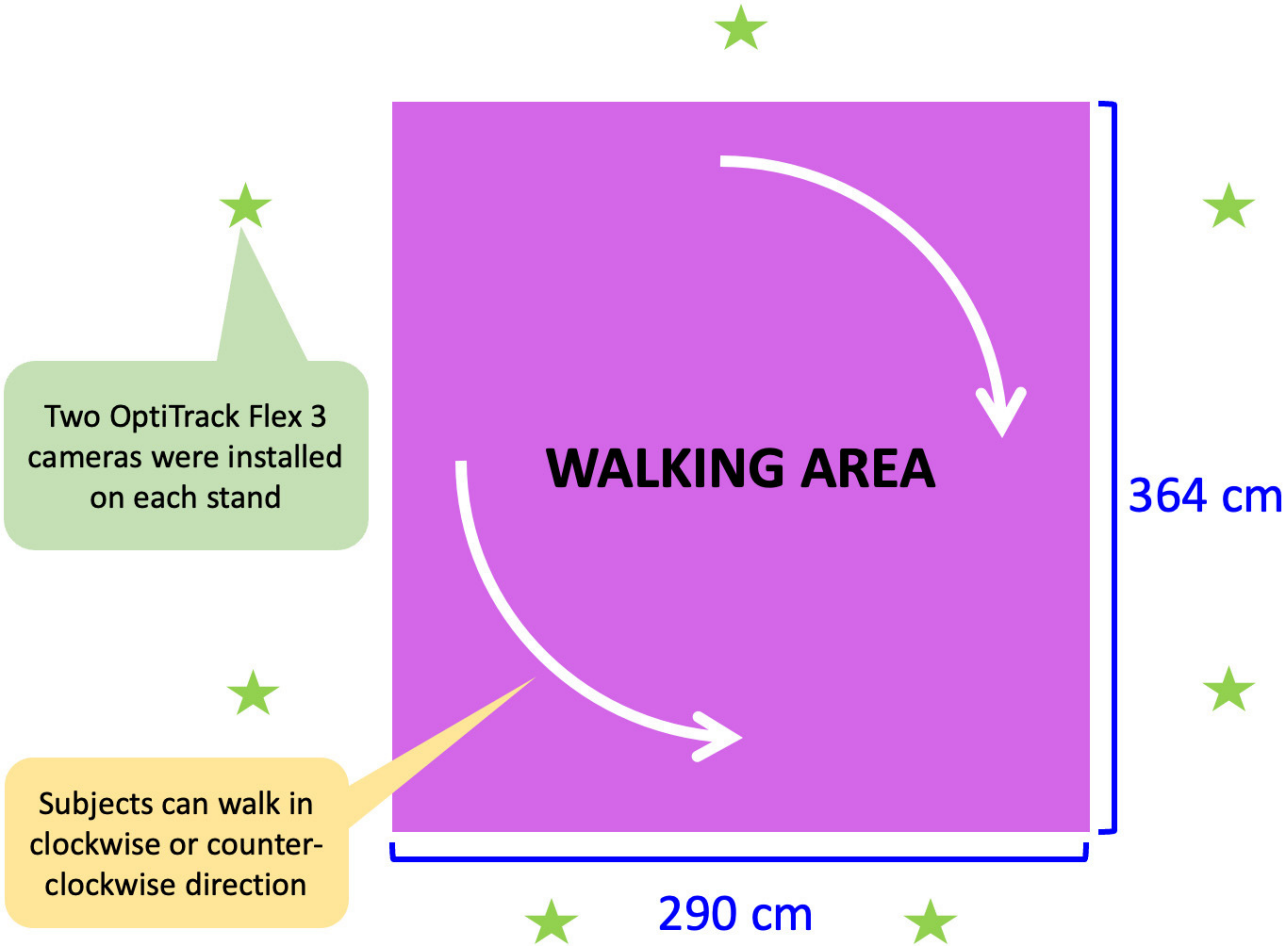
Dimensions of walking area and position of recording equipment (OptiTrack Flex 3).

We marked a rectangle on the floor for use as the walking area that can be captured by the OptiTrack motion tracking system, using black tape as shown in Figure 3. Fourteen OptiTrack Flex 3 motion-capture cameras were installed on seven camera stands. That is, two cameras were mounted on each stand at different heights as shown in Figure 2. The seven camera stands were placed around the walking area as illustrated in Figure 4. The size of the walking area is 2.9 by 3.64 m.

### 3.3. Materials for data collection

We selected three videos as stimuli for emotion induction. These videos were shown to the subjects using HoloLens 2 as they walked through the recording area.

- **Neutral video:**
  The nature landscape video from YouTube named *Spectacular drone shots of Iowa corn fields* uploaded by the YouTube user named *The American Bazaar*.^2^
- **Negative video:**
  An emotional movie selected from the *LIRIS-ACCEDE* database named *Parafundit* by *Riccardo Melato*.
- **Positive video:**
  An emotional movie selected from the *LIRIS-ACCEDE* database named *Tears of steel* by *Ian Hubert* and *Ton Roosendaal*.

A neutral video was selected from landscape videos on YouTube, based on the assumption that it would not induce any emotion.

Neutral video is the nature view of a corn field located in Des Moines, Iowa, USA. It was recorded by a drone camera so it contains the aerial view of the corn field which has mostly green color for the entire video. This video does not have any sound. Because of these reasons, we thought it would not induce any emotion. Positive (inducing happy emotion) and negative (inducing sad emotion) videos were selected from the public annotated movie database *LIRIS-ACCEDE*^3^ published by Baveye et al. (2015). This database contains many Creative Commons movies and their emotional annotations. In this study, we used the Continuous LIRIS-ACCEDE collection that contains 30 movies and emotion annotations in Valence-Arousal ranking. Most movies contain both positive and negative valence in the same movie. We carefully selected one movie that has positive valence for the entire movie and one movie that has negative valence for the entire movie to design a complete walking trial that contains only one emotion.

The negative video we selected is a short movie named *Parafundit*. This movie is the story of a man living alone. He commits suicide at the end of the movie using a gun. In addition to the valence score which is negative for the entire movie, we thought the mood and tone of this video are very suitable for inducing sadness. For positive video, we selected a movie named *Tears of steel* which is a short science fiction movie. The main idea of this movie is a group of scientists attempts to save the world from destructive robots. The attempt has been successful at the end of this movie. Therefore, we chose this movie as the movie to induce happiness as well as the valence score from the LIRIS-ACCEDE annotation is positive for the entire movie. We were also concerned about the length of each video, so we decided that none of the videos used would exceed 15 min in length. The lengths of the neutral video, negative movie, and positive movie are 5:04, 13:10, and 12:14 min, respectively. Audio of negative and positive videos contain music, sound effects, and conversation in English. Subjects could hear the videos' sound from the HoloLens 2 built-in speakers as they walked. The neutral video does not contain any sound to ensure that it does not induce any emotion.

### 3.4. Methods for data collection

**Figure 5:**
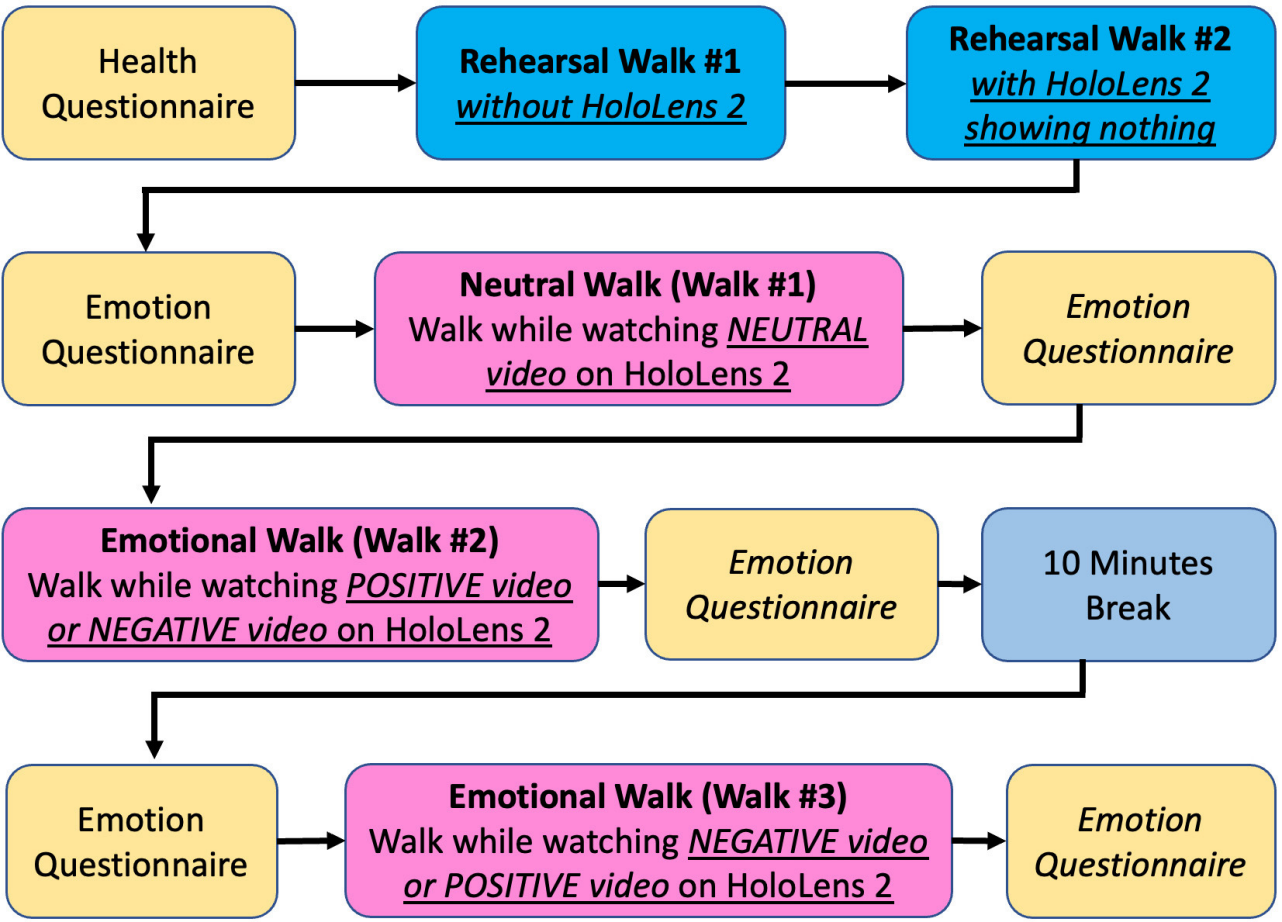
Data collection process.

The procedures of our data collection are shown in Figure 5. First, each participant was asked to answer the health questionnaire and sign the consent form before participating in our experiments. The health questionnaire consists of the following questions.

1. Do you have any neurological or mental disorders?
2. Do you have a severe level of anxiety or depression?
3. Do you have hearing impairment that cannot be corrected?
4. Do you have any permanent disability or bodily injury that affects your walking posture?
5. Do you feel sick now? (e.g., fever, headache, stomachache)
6. If you have any problem with your health condition, please describe it.

Based on the answers to this questionnaire, a subject could be excluded from participation in our experiment if he or she had any health issue. In this study, we found that all subjects were healthy, so all of them could participate in our experiment. After we confirmed that the subject was physically and mentally healthy, the subject was asked to walk in a circular pattern inside the recording area marked by black tape on the floor as shown in Figure 3. All participants could select the direction they wanted to walk, between clockwise or counter-clockwise, inside the walking area. In addition, the subjects could switch their walking direction from clockwise to counter-clockwise or vice versa whenever they wanted for an unlimited number of times.

Before performing the actual recording, each subject was asked to walk naturally in the recording area for 3 min without wearing HoloLens 2 as *Rehearsal Walk #1* to make the subject feel familiar with the walking space. Then, each subject was asked to wear the HoloLens 2 while it did not show anything and walk again for 3 min as *Rehearsal Walk #2* to make the subject feel familiar with walking while wearing HoloLens 2. In this walk, the HoloLens 2 is just a pair of transparent smart glasses showing no content at all. As we found in previous studies, including Kim et al. (2018), Sedighi et al. (2018), and Sedighi et al. (2020), if the participants never experienced using smart glasses while walking, adverse effects could occur and gait performance could be unstable. Our attempt to cope with this issue was to ask all subjects to take the rehearsal walks with and without wearing HoloLens 2 before performing actual recording.

To perform actual recording, the *Neutral Video* was displayed on HoloLens 2. Each subject was asked to walk and watch the video at the same time. The goal of this experiment is to capture a *Neutral Walk*. Participants started walking when the video started playing and stopped walking when the video ended. Then, we performed recording for the first *Emotional Walk* using a similar method as that used for *Neutral Walk*. In this experiment, each subject was asked to walk while watching the *Positive Video* or *Negative Video* selected from the LIRIS-ACCEDE database as mentioned in Section 3.3. After finishing the first *Emotional Walk*, each subject was asked to leave the experiment room to take a 10-min break as a reset of the induced emotion back to normal condition. After the break, we performed the second *Emotional Walk* experiment by showing another emotional video on HoloLens 2 while the subjects walked. If the first emotional walk was done using *Positive Video*, the second emotional walk was done using *Negative Video*. The order of Negative Walk and Positive Walk was swapped for the next subject. Hence, if the first emotional walk was done using *Negative Video*, the second emotional walk was done using *Positive Video*. Note that each subject was asked to answer the self-reported emotion questionnaire before and after walking and watching each video. These questions appeared as follows.

1. Please choose your current feeling: Happy, Sad, Neither (Not Sad and Not Happy)
2. How intense is your feeling: 1 (Very Little) to 5 (Very Much).

### 3.5. Collected dataset

There were 49 participants in this dataset: *41 male and 8 female* subjects. The average age of participants was 19.69 years. The standard deviation of participants' ages was 1.40 years. The average height was 168.49 cm. The standard deviation of height was 6.34 cm. The average weight was 58.88 kg, and the standard deviation of weight was 10.84 kg. The variance of subjects' ages was not large since all participants were undergraduate university students. We also made separate statistics for male and female subjects as follows.

**For female subjects:**

- Number of subjects: 8 participants
- Average/SD of *Age*: 19.25/0.89 years
- Average/SD of *Height*: 160.38/3.58 cm
- Average/SD of *Weight*: 51.25/3.28 kg.

**For male subjects:**

- Number of subjects: 41 participants
- Average/SD of *Age*: 19.78/1.47 years
- Average/SD of *Height*: 170.07/5.51 cm
- Average/SD of *Weight* 60.37/11.18 kg.

Each subject walked and watched three videos including *Neutral Video, Negative Video, and Positive Video*. In all, a total of 147 walking trials were conducted.

For the order of videos shown to the subjects, 24 subjects watched *Negative Video* before *Positive Video*, and 25 subjects watched *Positive Video* before *Negative Video*.

According to the answers from the self-reported emotion questionnaire completed after subjects finished walking and watching each video, we had 44 *Sad* walking trials, 44 *Happy* walking trials, and 59 *Neither* walking trials. These emotion tags *(Reported Emotion)* were used in our analysis instead of the emotion tags of the videos *(Expected Emotion)* because not all subjects felt *Happy* after watching *Positive Video* and not all subjects felt *Sad* after watching *Negative Video*. Table 2 shows the numbers of subjects who felt Happy, Sad, and Neither from the self-reported emotion questionnaire for each video stimulus.

**Table 2:** Comparison of expected emotion from stimuli and reported emotion from self-reported questionnaire.

**Stimuli\reported emotion**	**Happy**	**Sad**	**Neither**
Positive movie	12	23	14
Negative movie	13	19	17
Neutral movie	19	2	28

**Figure 6:**
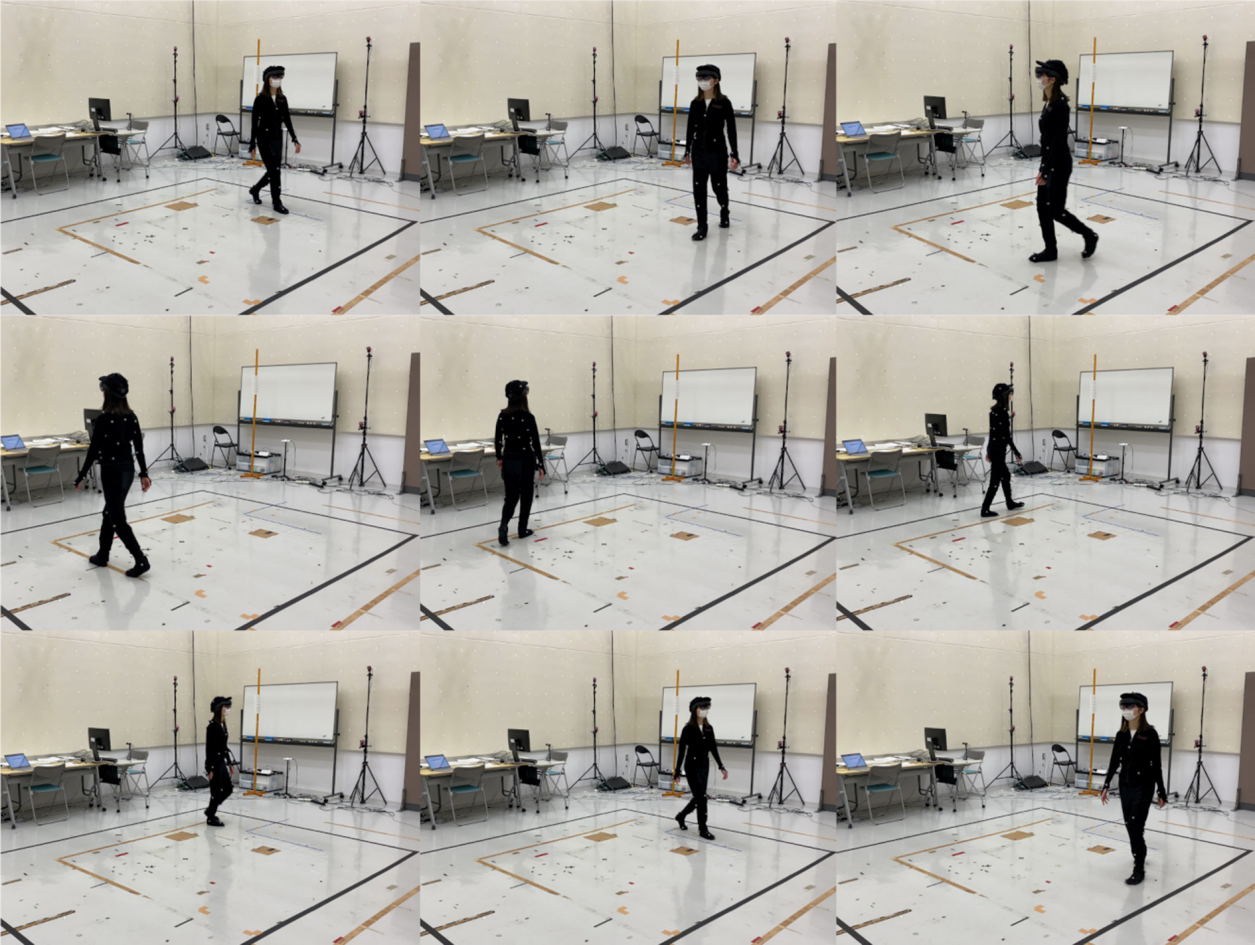
Sample images of walking subject while watching video on HoloLens 2.

**Figure 7:**
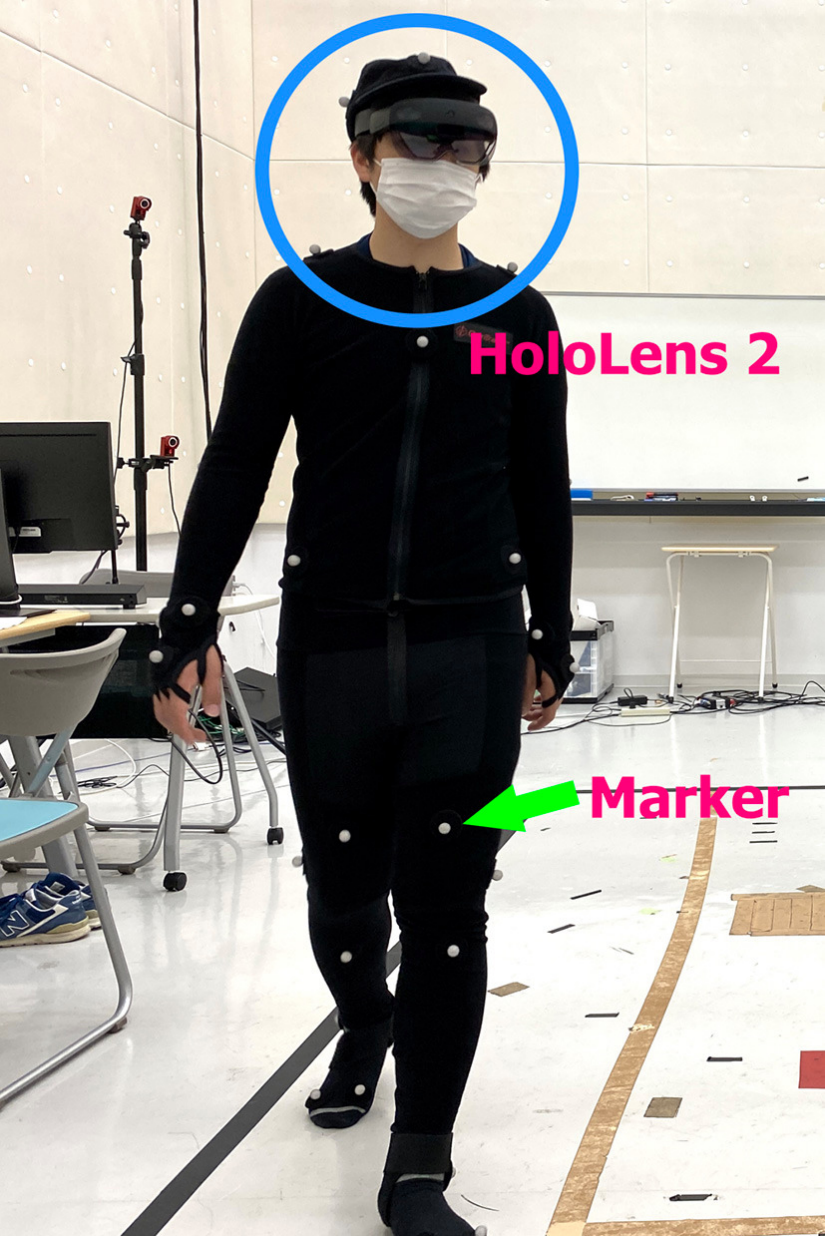
A subject wearing HoloLens 2 and OptiTrack motion capture suit with 37 markers.

Sample images of a subject walking in circular patterns in the recording area while watching a video on HoloLens 2 are shown in Figure 6, and a photo of a subject wearing the OptiTrack Motion Capture Suit with 37 markers and HoloLens 2 is shown in Figure 7.

From a total of 147 walking trials, one was corrupted during recording, so we had 146 usable walking trials. For the direction of walking, we had 99 counter-clockwise walking trials, 21 clockwise walking trials, and 26 walking trials with both clockwise and counter-clockwise directions in one walk.

## 4. Data preprocessing

After extracting 3-dimensional coordinates of 37 body markers captured by OptiTrack, we performed preprocessing steps to clean up the data and remove unusable data and noise as follows.

First, we removed 1 min from the beginning and 1 min from the end of each walking trial's data. Since the OptiTrack motion-capturing system can capture marker coordinate data at 100 frames per second, the first 6,000 frames and the last 6,000 frames from each walking trial were removed.

**Figure 8:**
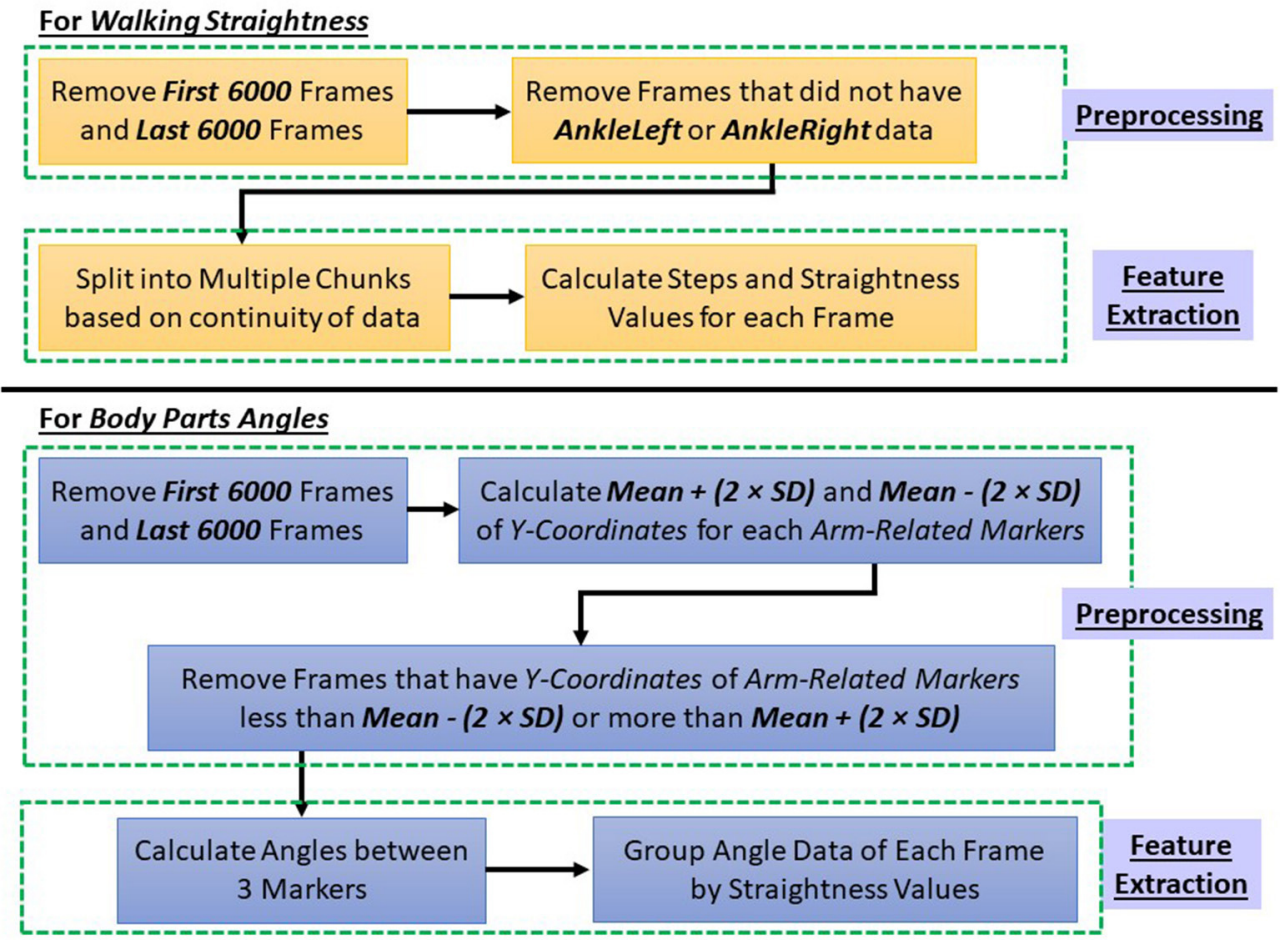
Preprocessing steps for straightness and body part angles.

In the feature extraction process, we extracted *Walking Straightness* and *Body Part Angles*. The feature extraction process is explained in Section 5. However, *Walking Straightness* and *Body Part Angles* have different preprocessing steps as shown in Figure 8. Therefore, the preprocessing steps of *Walking Straightness* and *Body Part Angles* are explained separately below.

After removing the first 6,000 and the last 6,000 frames, for *Walking Straightness* feature extraction, we used the position of *Left Foot* and *Right Foot*. Therefore, we checked each frame for whether the coordinate data of *LeftAnkleOut* and *RightAnkleOut* were available. If these two markers' data were missing in any frame, we excluded that frame from the straightness calculation.

For *Body Part Angles* feature extraction, in addition to removing the first and last 6,000 frames, we also removed any frame that contained arm movement that was not part of natural walking. Examples of these movements are when subjects raised their arms to check the time on their watch, subjects tried to adjust the position of the HoloLens 2 smart glasses, or subjects scratched their head while walking. This preprocessing step was done by checking the Y-coordinates of arm-related markers, i.e., left and right *HandOut, WristOut, WristIn, and ElbowOut*. For each arm-related marker, we calculated mean value and standard deviation value of its Y-coordinate for each walking trial. If the Y-coordinate in any frame was less than *Mean* − 2 × *SD* or more than *Mean* + 2 × *SD*, we removed that frame from the angle calculation.

## 5. Feature extraction

**Figure 9:**
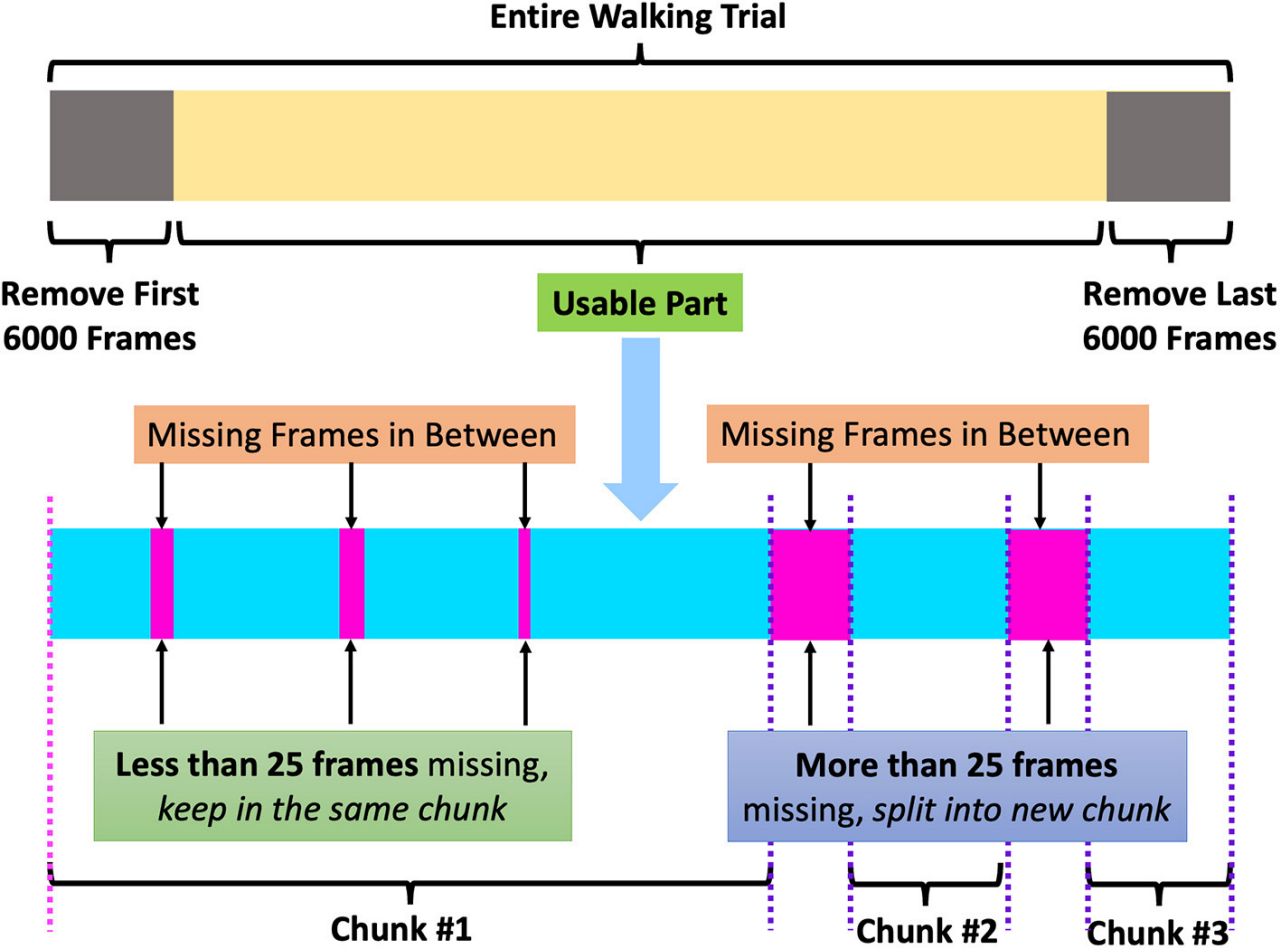
Splitting a walking trial into chunks for straightness and angle calculation.

After the preprocessing of coordinate data, there were some missing frames between the walking trials. This made the walking trial no longer continuous. Before we could proceed to the next step, we coped with this issue by splitting a walking trial into multiple chunks based on the missing frames. If there were more than 25 contiguous missing frames, we spit the trial into new walking chunks. If the length of missing frames was less than 25 frames, we still kept the next available frames in the same chunk. A diagram showing chunk splitting is given in Figure 9. However, if a chunk was smaller than 50 frames (0.5 s), we discarded that chunk since it was too small and not usable.

To calculate the straightness of a walking trial, we detected the steps of a walk first. For each chunk, we calculated distance between left foot and right foot in the top-view for all frames. That is, we calculated Euclidean distance between the *X* and *Z* coordinates of left foot and right foot. Then, we performed data smoothing by Savitzky-Golay filter before detecting the peaks of distance value from each chunk. By finding the peaks of distance between two feet, we could detect walking steps. If any chunk has less than three steps, we also discarded that chunk.

**Figure 10:**
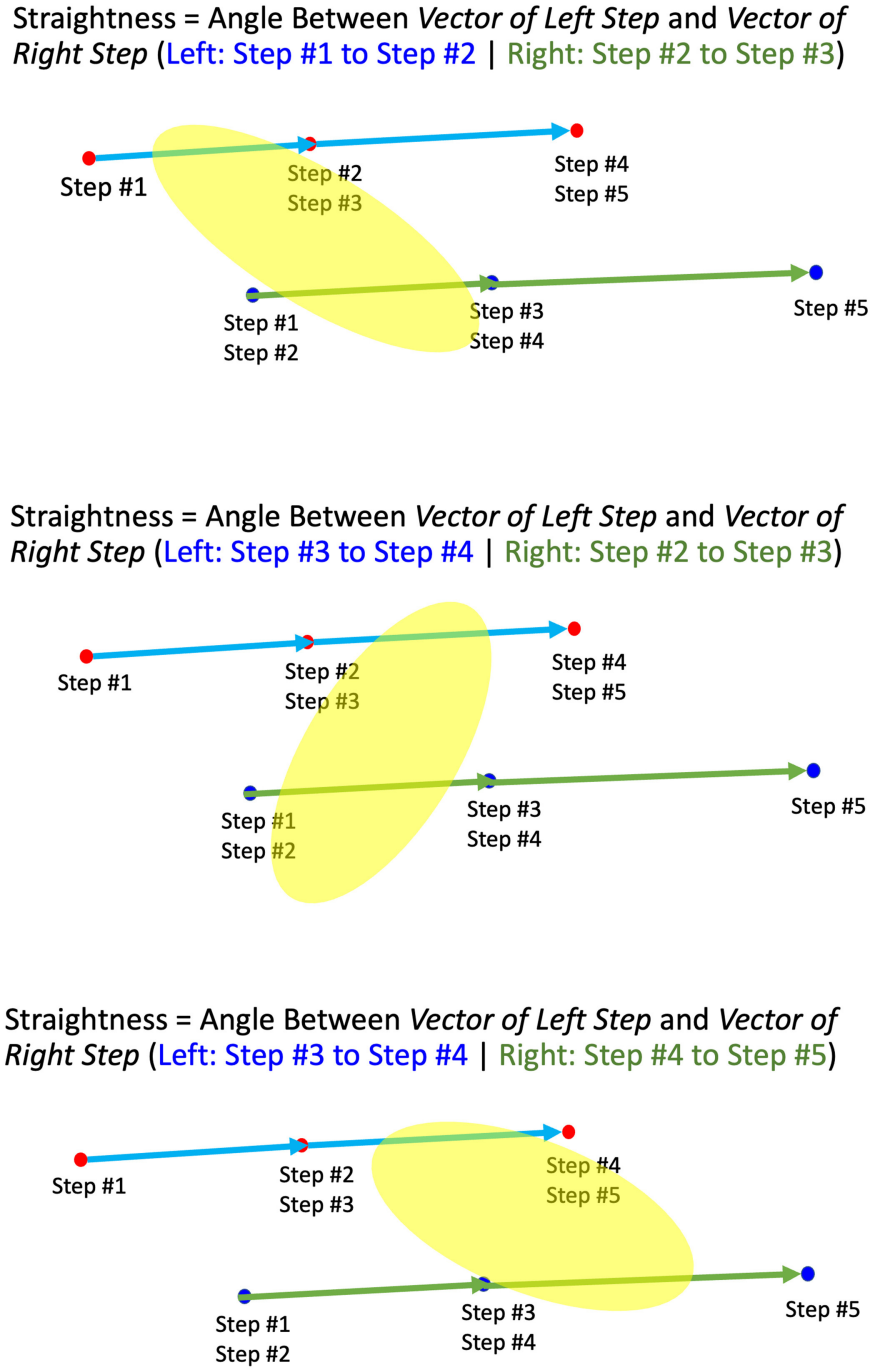
Finding angles between left and right vector to calculate straightness.

We calculated straightness of walking in each chunk by using three consecutive walking steps. An example diagram of straightness calculation from three consecutive steps is shown in Figure 10. In this figure, *Step #1* to *Step #2* is left step, and *Step #2* to *Step #3* is right step. Therefore, the first straightness value for *Step #1* to *Step #3* is the angle between the vector of left step (*Step #1* to *Step #2*) and right step (*Step #2* to *Step #3*). The detailed process of straightness calculation is as follows.

1. If *Step #1* to *Step #2* is left step, define the left step vector *v*_*left*12_ as a vector from (*X, Z*) point of *Step #1* to (*X, Z*) point of *Step #2*
2. Define the right step vector *v*_*right*23_ as a vector from (*X, Z*) point of *Step #2* to (*X, Z*) point of *Step #3*
3. **Calculate angle** **θ_13_** **of** ***v*_*left*12_** **to** ***v*_*right*23_**
4. Define the next left step vector *v*_*left*34_ as a vector from (*X, Z*) point of *Step #3* to (*X, Z*) point of *Step #4*
5. **Calculate angle** **θ_24_** **of** ***v*_*left*34_** **to** ***v*_*right*23_**
6. Define the next right step vector *v*_*right*45_ as a vector from (*X, Z*) point of *Step #4* to (*X, Z*) point of *Step #5*
7. **Calculate angle** **θ_35_** **of** ***v*_*left*34_** **to** ***v*_*right*45_**
8. If *Step #1* to *Step #2* is right step, the vector from (*X, Z*) point of *Step #1* to (*X, Z*) point of *Step #2* is *v*_*right*12_, and the vector from (*X, Z*) point of *Step #2* to (*X, Z*) point of *Step #3* is *v*_*left*23_. This rule applies to all consecutive steps, i.e., θ_13_ is angle between *v*_*right*12_ and *v*_*left*23_, θ_24_ is angle between *v*_*right*34_ and *v*_*left*23_ and θ_35_ is angle between *v*_*right*34_ and *v*_*left*45_.
9. Continue calculating the angle between left and right vector until each chunk is finished
10. Angle between left and right step vector is used as straightness value of the frames having these steps.

After obtaining the walking straightness of each of the three consecutive steps, we assigned the straightness values to all frames consisting of the corresponding steps. Now all frames have their own straightness values. Then, for each frame, the angles between the three body parts' positions were calculated. These include, for example, the angle between *LeftShoulderBack* and *LeftUpperArmHigh* and *LeftShoulderBack* and *BackLeft*, the angle between *BackTop* and *BackLeft* and *BackTop* and *HeadTop*, and so forth. In total, we calculated 24 angles from each of the three markers. All angles we used in this study are listed in Table 3.

**Table 3:** List of angles for each group of 3 markers.

**Angle index**	**Terminal point #1**	**Initial point**	**Terminal point #2**
1	LeftAnkleOut	LeftKneeOut	WaistLeftFront
2	RightAnkleOut	RightKneeOut	WaistRightFront
3	LeftShin	LeftKneeOut	LeftThigh
4	RightShin	RightKneeOut	RightThigh
5	LeftKneeOut	LeftThigh	WaistLeftFront
6	RightKneeOut	RightThigh	WaistRightFront
7	LeftThigh	WaistLeftFront	Chest
8	RightThigh	WaistRightFront	Chest
9	WaistLeftFront	Chest	LeftShoulderTop
10	WaistRightFront	Chest	RightShoulderTop
11	LeftKneeOut	WaistLeftBack	BackLeft
12	RightKneeOut	WaistRightBack	BackRight
13	WaistLeftBack	BackLeft	LeftShoulderBack
14	WaistRightBack	BackRight	RightShoulderBack
15	LeftShoulderBack	BackTop	HeadTop
16	RightShoulderBack	BackTop	HeadTop
17	BackLeft	BackTop	HeadTop
18	BackRight	BackTop	HeadTop
19	BackLeft	LeftShoulderBack	LeftUpperArmHigh
20	BackRight	RightShoulderBack	RightUpperArmHigh
21	BackTop	LeftShoulderBack	LeftUpperArmHigh
22	BackTop	RightShoulderBack	RightUpperArmHigh
23	LeftUpperArmHigh	LeftElbowOut	LeftWristOut
24	RightUpperArmHigh	RightElbowOut	RightWristOut

Next, a frame that consists of 24 angles was grouped into seven straightness groups based on the straightness value of that frame. Our straightness groups include one group for straight walking and six groups for curved walking as listed below. Every frame in a walking trial was grouped using its straightness value.

Because we used three consecutive steps to calculate a straightness value, the frames of connecting steps for each group of three consecutive steps had two straightness values, and in this case, the average of the two straightness values for those frames was used. For example, the straightness value of *Step #1* to *Step #3* is the angle between *Step #1* and *Step #2* and ***Step #2* and** ***Step #3***, and the straightness value of *Step #2* to *Step #4* is the angle between ***Step #2* and** ***Step #3*** and *Step #3* and *Step #4*; in this case, the frames of ***Step #2* to** ***Step #3*** had two straightness values. Therefore, the average straightness value of *Step #1* to *Step #3* and *Step #2* to *Step #4* was assigned to those frames.

After grouping frames into seven straightness groups, the mean value and standard deviation value of each angle in each group were calculated for each walking trial.

−35° to −25° (Large Curved Walking - Clockwise)
−25° to −15° (Moderate Curved Walking - Clockwise)
−15° to −5° (Small Curved Walking - Clockwise)
−5° to 5° (Straight Walking)
- 5° to 15° (Small Curved Walking - Counter-Clockwise)
- 15° to 25° (Moderate Curved Walking - Counter-Clockwise)
- 25° to 35° (Large Curved Walking - Counter-Clockwise).

**Figure 11:**
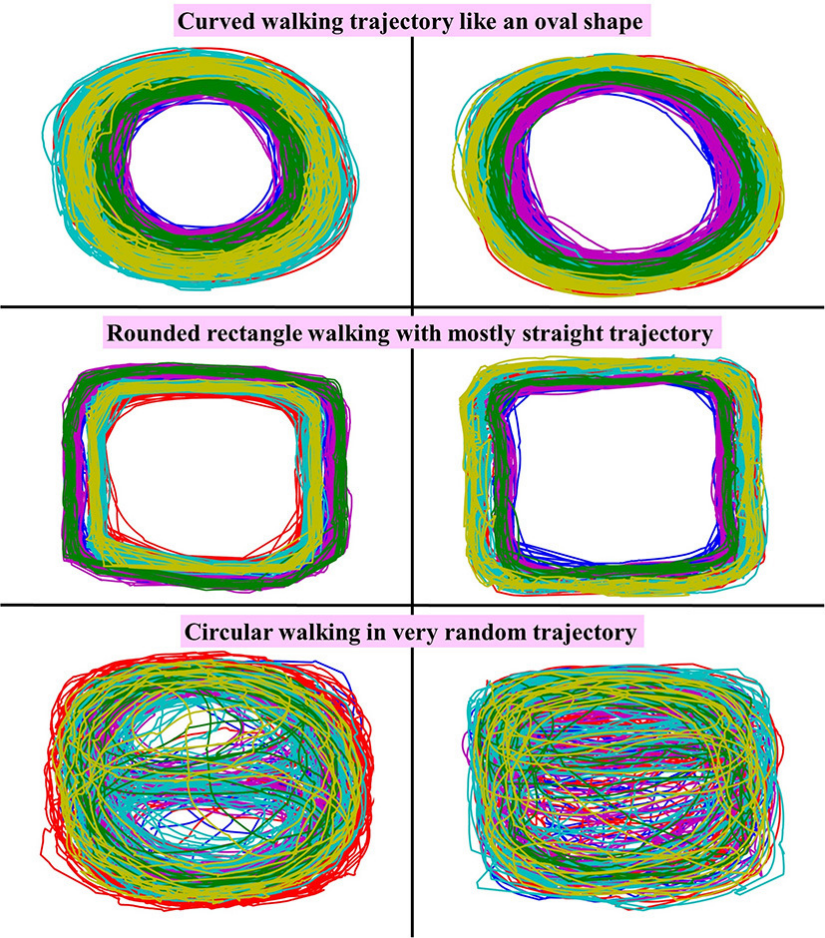
Sample of walking path from six subjects in this dataset, including oval-shaped walking, rounded-rectangular walking, and random circular walking.

We also checked the walking direction of each walking trial, since subjects were instructed to walk in a circular path inside the recording area but there was no designated path. Each subject chose his or her own path to walk inside the recording area, and we found that some subjects walked in an extremely curved pattern while other subjects walked in a very straight pattern. Consequently, there were multiple walking curvatures in one walking trial, e.g., straight walking part and non-straight (curved) walking part. Samples of walking trajectories for six subjects are shown in Figure 11, which reveals various walking trajectories. The walking paths of the first two subjects are curvy and closer to an oval shape, whereas the walking paths of the next two subjects are very straight for most of the parts, with some curved part only when they turned. These walking paths look more like a rounded-rectangle shape. However, for the last two subjects, their walking paths were very random but still in a circular pattern. In other words, these two subjects walked in a very random circle size with random trajectories. According to our dataset, we found that the walking paths of most participants are quite regular like the first four examples. Very few subjects had highly random walking paths like the last two examples. Therefore, our dataset consists of many walking trajectories and curvatures. We can say that our dataset is *direction-free*, since the participants had the freedom to choose their walking path as they wished between clockwise and counter-clockwise directions.

## 6. Statistical analysis of gait features

The following analyses were performed on the mean and standard deviations of each angle.

### 6.1. One-way analysis of variance (1-way ANOVA)

#### 6.1.1. Methodology

We performed 1-way ANOVA to check whether emotion differences affect the movements of each body part. Both *Expected Emotion*, which is the emotion label from annotated videos we used as stimuli, and *Reported Emotion*, which is the emotion label from self-reported questionnaire answers, were used in this analysis.

- **Factor to test:** Expected Emotion (Positive, Negative, Neutral) and Reported Emotion (Happy, Sad, Neither)
- **Dependent variable:** Mean value and SD value of each angle in each straightness group.

Mean and SD values of all angles for the two types of emotions, Expected Emotion and Reported Emotion, were compared separately according to their straightness groups in this analysis.

#### 6.1.2. Results

One-way ANOVA was used for checking the effects of *Expected Emotions* and *Reported Emotions* on the movements of body parts. Detailed results of the ANOVA test on mean and SD of each angle in each straightness group are shown in Table 4 for Expected Emotion and Table 5 for Reported Emotion. If the mean or SD value of any angle in any straightness group is significantly different among emotions, we performed a Tukey test with that mean or SD of that angle to find the pair of emotions that has significant effects on body movements, e.g., Happy vs. Sad or Neither vs. Sad. Tukey test results for Expected Emotion and Reported Emotion are shown in Table 6.

**Table 4:** Results of 1-way ANOVA for mean and SD of each angle in each straightness group (factor: expected emotion).

**Angle name**	**Type**	**Degrees of** **freedom**	−**35°** **to** −**25°**		−**25°** **to** −**15°**		−**15°** **to** −**5°**		−**5°** **to 5°**		**5°** **to 15°**		**15°** **to 25°**		**25°** **to 35°**	

			***F*** **-value**	***P*** **-value**	***F*** **-value**	***P*** **-value**	***F*** **-value**	***P*** **-value**	***F*** **-value**	***P*** **-value**	***F*** **-value**	***P*** **-value**	***F*** **-value**	***P*** **-value**	***F*** **-value**	***P*** **-value**
LeftAnkleOut-LeftKneeOut-WaistLeftFront	Mean	2	0.6987	0.5019	0.0630	0.9390	0.5669	0.5691	0.4628	0.6305	0.7150	0.4911	0.4281	0.6527	0.0274	0.9730
	SD	2	1.4229	0.2503	0.7884	0.4596	0.2048	0.8151	1.1753	0.3119	0.7593	0.4701	0.6692	0.5140	0.0619	0.9400
RightAnkleOut-RightKneeOut-WaistRightFront	Mean	2	0.0400	0.9608	0.2012	0.8183	0.2380	0.7887	0.2609	0.7707	0.1876	0.8292	0.0528	0.9486	0.1438	0.8662
	SD	2	0.3288	0.7213	0.5030	0.6074	0.5824	0.5604	0.4580	0.6335	0.3936	0.6754	0.2081	0.8124	0.3454	0.7086
LeftShin-LeftKneeOut-LeftThigh	Mean	2	0.3689	0.6933	0.0266	0.9738	0.0402	0.9606	0.1281	0.8799	0.0466	0.9545	0.0124	0.9877	0.0645	0.9376
	SD	2	0.9747	0.3841	0.8820	0.4196	0.0043	0.9957	0.1084	0.8973	0.2077	0.8127	0.1842	0.8320	0.1457	0.8645
RightShin-RightKneeOut-RightThigh	Mean	2	0.0961	0.9086	0.5365	0.5878	0.1823	0.8336	0.0654	0.9367	0.2214	0.8017	0.0934	0.9109	0.0191	0.9811
	SD	2	0.2366	0.7901	0.7163	0.4930	0.2288	0.7959	0.4617	0.6312	0.2421	0.7854	0.0345	0.9661	0.2518	0.7778
LeftKneeOut-LeftThigh-WaistLeftFront	Mean	2	0.1162	0.8905	0.0672	0.9351	0.3171	0.7290	0.1885	0.8285	0.1517	0.8594	0.1323	0.8762	0.0731	0.9296
	SD	2	0.3972	0.6742	0.5685	0.5696	0.1123	0.8939	0.1519	0.8592	0.0871	0.9167	0.2593	0.7720	0.1770	0.8380
RightKneeOut-RightThigh-WaistRightFront	Mean	2	0.0272	0.9732	0.2478	0.7814	0.3318	0.7184	0.0409	0.9599	0.0489	0.9523	0.0056	0.9944	0.0342	0.9664
	SD	2	0.0176	0.9826	0.4165	0.6614	0.0561	0.9455	0.5370	0.5857	0.1358	0.8732	0.0396	0.9612	0.2599	0.7716
LeftThigh-WaistLeftFront-Chest	Mean	2	0.0575	0.9442	0.2484	0.7809	0.0225	0.9777	0.0505	0.9508	0.0442	0.9568	0.0635	0.9385	0.0796	0.9235
	SD	2	0.7141	0.4944	0.7336	0.4847	0.0286	0.9719	0.0578	0.9439	0.5089	0.6023	0.4121	0.6632	0.2039	0.8158
RightThigh-WaistRightFront-Chest	Mean	2	0.0019	0.9981	0.0953	0.9093	0.3291	0.7204	0.0359	0.9647	0.0404	0.9605	0.0015	0.9985	0.0004	0.9996
	SD	2	0.2227	0.8011	0.3880	0.6802	0.6301	0.5346	0.2490	0.7799	0.3234	0.7243	0.5774	0.5629	0.7637	0.4682
WaistLeftFront-Chest-LeftShoulderTop	Mean	2	0.0673	0.9350	0.1955	0.8230	0.0322	0.9684	0.0839	0.9196	0.0410	0.9598	0.0633	0.9387	0.0654	0.9367
	SD	2	0.4684	0.6286	1.2575	0.2923	0.4890	0.6147	0.4218	0.6567	0.6656	0.5157	0.6061	0.5471	0.8730	0.4203
WaistRightFront-Chest-RightShoulderTop	Mean	2	0.0326	0.9679	0.0055	0.9945	0.1610	0.8515	0.0369	0.9638	0.0259	0.9744	0.1441	0.8660	0.1333	0.8753
	SD	2	0.9598	0.3897	1.2461	0.2955	0.3207	0.7264	0.1894	0.8277	0.2634	0.7688	0.1474	0.8631	0.4252	0.6546
LeftKneeOut-WaistLeftBack-BackLeft	Mean	2	0.1133	0.8931	0.0771	0.9259	0.4082	0.6660	0.3419	0.7110	0.0088	0.9913	0.0080	0.9920	0.0221	0.9782
	SD	2	0.6723	0.5149	0.7105	0.4958	0.1871	0.8296	0.1079	0.8978	0.2782	0.7576	0.1335	0.8752	0.0226	0.9776
RightKneeOut-WaistRightBack-BackRight	Mean	2	0.0326	0.9680	1.0470	0.3577	0.0834	0.9200	0.5913	0.5550	1.0922	0.3385	0.5290	0.5906	0.3380	0.7139
	SD	2	0.4738	0.6253	0.3182	0.7287	0.8494	0.4307	1.9243	0.1500	1.9279	0.1496	1.1144	0.3314	0.2577	0.7732
WaistLeftBack-BackLeft-LeftShoulderBack	Mean	2	0.0055	0.9945	0.0524	0.9490	0.0588	0.9430	0.1206	0.8864	0.1133	0.8930	0.1484	0.8622	0.1787	0.8366
	SD	2	0.2455	0.7832	0.0746	0.9282	0.1165	0.8902	0.5529	0.5766	0.3701	0.6914	0.1270	0.8808	0.2624	0.7696
			***F*** **-value**	***P*** **-value**	***F*** **-value**	***P*** **-value**	***F*** **-value**	***P*** **-value**	***F*** **-value**	***P*** **-value**	***F*** **-value**	***P*** **-value**	***F*** **-value**	***P*** **-value**	***F*** **-value**	***P*** **-value**
WaistRightBack-BackRight-RightShoulderBack	Mean	2	0.0164	0.9837	0.2105	0.8108	0.2754	0.7599	0.1907	0.8266	0.4627	0.6306	0.1733	0.8411	0.1627	0.8501
	SD	2	0.6988	0.5018	0.8341	0.4396	0.7722	0.4647	1.9541	0.1457	1.8371	0.1634	1.8335	0.1643	0.9480	0.3904
LeftShoulderBack-BackTop-HeadTop	Mean	2	0.1003	0.9048	0.0646	0.9375	0.0058	0.9942	0.0909	0.9131	0.1221	0.8851	0.0539	0.9475	0.0722	0.9304
	SD	2	0.5386	0.5868	0.1024	0.9028	0.6025	0.5494	1.8318	0.1641	1.9491	0.1465	2.0333	0.1353	2.4667	0.0891
**RightShoulderBack-BackTop-HeadTop**	Mean	2	0.2849	0.7533	0.3381	0.7146	0.1859	0.8307	0.7313	0.4832	0.4392	0.6455	0.3593	0.6989	0.3400	0.7125
	**SD**	2	0.3272	0.7224	0.0465	0.9546	1.6002	0.2069	2.3775	0.0967	1.5533	0.2154	1.8237	0.1658	3.6983	**0.0276**
BackLeft-BackTop-HeadTop	Mean	2	0.1957	0.8229	0.1944	0.8239	0.2522	0.7776	0.3959	0.6739	0.4448	0.6419	0.2402	0.7869	0.2648	0.7678
	SD	2	0.9232	0.4037	0.2502	0.7795	0.1858	0.8307	1.7377	0.1799	1.1061	0.3339	1.1375	0.3240	1.0224	0.3628
BackRight-BackTop-HeadTop	Mean	2	0.1226	0.8849	0.1470	0.8637	0.1691	0.8447	0.0811	0.9221	0.3629	0.6964	0.2208	0.8022	0.3136	0.7314
	SD	2	1.2557	0.2934	0.1276	0.8805	0.6979	0.5000	1.3334	0.2670	1.4269	0.2438	1.9193	0.1511	1.3109	0.2734
BackLeft-LeftShoulderBack-LeftUpperArmHigh	Mean	2	1.0053	0.3730	0.7051	0.4984	0.6048	0.5482	0.0536	0.9478	0.0696	0.9328	0.1110	0.8951	0.0803	0.9229
	SD	2	0.8814	0.4203	1.2342	0.2989	0.7095	0.4943	0.8023	0.4505	0.6222	0.5384	0.8106	0.4470	1.5265	0.2215
BackRight-RightShoulderBack-RightUpperArmHigh	Mean	2	0.1877	0.8294	0.1031	0.9022	0.7849	0.4590	0.4870	0.6156	0.2734	0.7612	0.2687	0.7648	0.1749	0.8397
	SD	2	1.0834	0.3460	1.3641	0.2640	0.4045	0.6684	0.5204	0.5955	0.5944	0.5534	0.4766	0.6220	0.4526	0.6370
BackTop-LeftShoulderBack-LeftUpperArmHigh	Mean	2	0.8956	0.4146	0.8612	0.4282	0.2046	0.8153	0.2388	0.7879	0.1776	0.8375	0.1046	0.9007	0.1015	0.9036
	SD	2	0.4737	0.6254	0.4183	0.6602	0.1621	0.8506	1.0618	0.3487	1.5991	0.2060	1.4489	0.2389	0.4938	0.6115
BackTop-RightShoulderBack-RightUpperArmHigh	Mean	2	0.3612	0.6986	0.4943	0.6126	0.2053	0.8148	0.7697	0.4652	0.5401	0.5840	0.3806	0.6843	0.3361	0.7152
	SD	2	0.3637	0.6969	0.7683	0.4686	0.0051	0.9949	0.5426	0.5825	0.6985	0.4992	1.1292	0.3267	1.9275	0.1500
LeftUpperArmHigh-LeftElbowOut-LeftWristOut	Mean	2	1.4454	0.2450	0.6532	0.5243	0.8158	0.4452	0.5184	0.5967	0.4014	0.6702	0.4382	0.6462	0.3603	0.6982
	SD	2	0.2067	0.8139	0.3699	0.6925	0.5952	0.5533	0.6584	0.5194	0.8055	0.4491	1.0016	0.3703	1.0944	0.3380
RightUpperArmHigh-RightElbowOut-RightWristOut	Mean	2	0.2920	0.7480	0.5579	0.5756	0.1339	0.8748	0.1503	0.8606	0.0079	0.9922	0.7635	0.4683	1.0489	0.3535
	SD	2	0.5626	0.5731	1.2252	0.3015	0.8732	0.4208	0.9758	0.3796	1.1729	0.3127	0.9895	0.3747	0.5837	0.5594

**Table 5:** Results of 1-way ANOVA for mean and SD of each angle in each straightness group (factor: reported emotion).

**Angle name**	**Type**	**Degrees of** **freedom**	−**35°** **to** −**25°**		−**25°** **to** −**15°**		−**15°** **to** −**5°**		−**5°to 5°**		**5°** **to 15°**		**15°** **to 25°**		**25°** **to 35°**	

			***F*** **-value**	***P*** **-value**	***F*** **-value**	***P*** **-value**	***F*** **-value**	***P*** **-value**	***F*** **-value**	***P*** **-value**	***F*** **-value**	***P*** **-value**	***F*** **-value**	***P*** **-value**	***F*** **-value**	***P*** **-value**
LeftAnkleOut-LeftKneeOut-WaistLeftFront	Mean	2	0.4845	0.6188	0.4657	0.6301	0.4470	0.6408	0.4713	0.6252	0.1020	0.9031	0.0909	0.9131	0.7818	0.4599
	SD	2	0.6812	0.5105	1.3633	0.2642	0.1778	0.8373	0.4065	0.6668	0.8391	0.4344	2.5143	0.0851	0.8202	0.4428
**RightAnkleOut-RightKneeOut-WaistRightFront**	**Mean**	2	0.5185	0.5985	5.5611	**0.0063**	2.9376	0.0575	0.2956	0.7446	0.3023	0.7396	0.0331	0.9674	0.0279	0.9725
	SD	2	0.5383	0.5869	0.2925	0.7476	1.3475	0.2645	0.6572	0.5200	0.7174	0.4899	1.9441	0.1476	0.8614	0.4251
LeftShin-LeftKneeOut-LeftThigh	Mean	2	0.0484	0.9528	0.2797	0.7571	0.0162	0.9839	0.3218	0.7254	0.5134	0.5996	0.5020	0.6066	1.0129	0.3662
	SD	2	1.0287	0.3646	0.8801	0.4204	2.2178	0.1141	0.8895	0.4133	1.5201	0.2226	2.3493	0.0998	0.7299	0.4841
RightShin-RightKneeOut-RightThigh	Mean	2	0.0336	0.9670	0.3826	0.6838	0.2186	0.8041	0.4932	0.6118	0.3929	0.6759	0.4171	0.6599	0.8981	0.4100
	SD	2	2.0007	0.1455	1.9058	0.1582	1.3421	0.2659	0.2658	0.7670	0.2274	0.7970	0.6056	0.5474	1.7291	0.1818
LeftKneeOut-LeftThigh-WaistLeftFront	Mean	2	0.3220	0.7262	0.1940	0.8242	0.0450	0.9560	0.9174	0.4021	0.6264	0.5361	0.7198	0.4889	0.6020	0.5493
	SD	2	0.2257	0.7988	1.0393	0.3604	2.6192	0.0778	0.3334	0.7171	0.2672	0.7659	0.7239	0.4869	0.1148	0.8916
RightKneeOut-RightThigh-WaistRightFront	Mean	2	0.0146	0.9855	0.2310	0.7945	0.3152	0.7303	0.7140	0.4915	0.5416	0.5831	1.1918	0.3072	0.9561	0.3873
	SD	2	0.1855	0.8313	1.2099	0.3059	0.1851	0.8313	0.9210	0.4006	0.4243	0.6551	1.4454	0.2397	1.2993	0.2765
LeftThigh-WaistLeftFront-Chest	Mean	2	0.3876	0.6807	0.0256	0.9747	0.8356	0.4366	1.1208	0.3291	0.9014	0.4085	0.9257	0.3991	0.8437	0.4326
	SD	2	0.2137	0.8083	0.7045	0.4987	1.6209	0.2028	1.6481	0.1963	0.4574	0.6340	0.5656	0.5695	0.3365	0.7149
RightThigh-WaistRightFront-Chest	Mean	2	0.1644	0.8488	0.3378	0.7148	0.9859	0.3767	0.4663	0.6283	0.1492	0.8616	0.7130	0.4922	0.4879	0.6151
	SD	2	0.8236	0.4445	2.5884	0.0841	0.6103	0.5452	1.8801	0.1566	0.5680	0.5680	0.1314	0.8770	0.2727	0.7618
**WaistLeftFront-Chest-LeftShoulderTop**	Mean	2	0.9145	0.4071	0.8174	0.4468	1.3427	0.2658	1.6144	0.2029	1.8164	0.1667	1.1176	0.3304	1.1843	0.3095
	**SD**	2	2.1649	0.1250	3.5329	**0.0359**	0.6072	0.5469	0.5502	0.5781	1.0983	0.3365	0.9759	0.3798	1.9595	0.1454
**WaistRightFront-Chest-RightShoulderTop**	Mean	2	0.7710	0.4677	0.6719	0.5148	0.4229	0.6563	0.2794	0.7567	0.3841	0.6818	0.7168	0.4904	0.8103	0.4471
	**SD**	2	2.0888	0.1341	4.6688	**0.0133**	0.6010	0.5502	1.9995	0.1394	0.4906	0.6134	1.3305	0.2682	1.5063	0.2259
**LeftKneeOut-WaistLeftBack-BackLeft**	Mean	2	0.2721	0.7629	0.3625	0.6976	0.2912	0.7480	1.8402	0.1628	1.0133	0.3659	0.7293	0.4844	0.7647	0.4677
	**SD**	2	0.2765	0.7596	3.4477	**0.0387**	0.7650	0.4680	0.5142	0.5992	0.8033	0.4501	1.5869	0.2088	1.0004	0.3707
RightKneeOut-WaistRightBack-BackRight	Mean	2	1.1541	0.3233	0.8457	0.4347	1.3287	0.2695	1.9146	0.1514	1.6376	0.1984	1.5744	0.2113	1.0199	0.3637
	SD	2	0.1448	0.8655	1.0217	0.3666	0.4833	0.6182	0.0701	0.9323	0.0521	0.9493	0.1252	0.8825	0.1395	0.8699
WaistLeftBack-BackLeft-LeftShoulderBack	Mean	2	0.1492	0.8617	0.3764	0.6881	0.2840	0.7534	1.0613	0.3489	0.9299	0.3972	1.0790	0.3432	0.9948	0.3728
	SD	2	0.1619	0.8510	0.2335	0.7925	0.7214	0.4886	0.3195	0.7271	2.0702	0.1303	3.1031	0.0485	2.4005	0.0950
WaistRightBack-BackRight-RightShoulderBack	Mean	2	0.7306	0.4865	0.9563	0.3905	0.4072	0.6666	2.4226	0.0926	2.1401	0.1218	2.1141	0.1252	2.1851	0.1169
	SD	2	0.6572	0.5225	0.8903	0.4163	0.8087	0.4483	0.2036	0.8161	0.0944	0.9100	0.3177	0.7284	0.4623	0.6309
LeftShoulderBack-BackTop-HeadTop	Mean	2	1.1209	0.3337	0.9886	0.3785	2.1281	0.1244	1.4770	0.2320	1.6585	0.1944	2.4785	0.0881	2.5454	0.0826
	SD	2	0.1825	0.8338	0.0951	0.9094	0.6739	0.5120	0.3303	0.7193	0.0124	0.9877	0.6777	0.5097	0.7725	0.4641
**RightShoulderBack-BackTop-HeadTop**	**Mean**	2	1.3644	0.2645	1.7783	0.1783	1.8622	0.1606	3.5905	0.0303	3.1532	0.0460	3.8224	**0.0246**	3.6483	**0.0289**
			***F*** **-value**	***P*** **-value**	***F*** **-value**	***P*** **-value**	***F*** **-value**	***P*** **-value**	***F*** **-value**	***P*** **-value**	***F*** **-value**	***P*** **-value**	***F*** **-value**	***P*** **-value**	***F*** **-value**	***P*** **-value**
	SD	2	0.0176	0.9825	0.1755	0.8395	0.3759	0.6877	0.1106	0.8954	0.0546	0.9469	1.2251	0.2973	1.3075	0.2743
BackLeft-BackTop-HeadTop	Mean	2	0.7225	0.4903	1.2970	0.2814	1.5026	0.2275	0.3595	0.6987	0.8208	0.4424	1.0128	0.3663	1.0605	0.3495
	SD	2	0.1865	0.8304	0.0673	0.9350	1.0389	0.3576	0.2770	0.7585	0.4246	0.6550	0.1453	0.8649	0.2800	0.7563
BackRight-BackTop-HeadTop	Mean	2	0.3262	0.7232	0.8130	0.4487	1.5697	0.2132	2.0942	0.1272	1.9272	0.1497	1.4560	0.2372	1.6514	0.1961
	SD	2	0.2449	0.7837	0.1288	0.8794	0.2129	0.8086	0.1499	0.8610	0.1021	0.9030	0.3622	0.6969	0.4358	0.6478
**BackLeft-LeftShoulderBack-LeftUpperArmHigh**	Mean	2	0.1312	0.8773	0.0324	0.9682	0.2414	0.7860	0.2599	0.7716	0.0830	0.9204	0.0986	0.9061	0.1756	0.8391
	**SD**	2	4.0633	**0.0229**	6.5990	**0.0027**	3.1264	**0.0481**	3.6658	**0.0282**	3.6448	**0.0288**	5.1320	**0.0073**	6.9080	**0.0014**
BackRight-RightShoulderBack-RightUpperArmHigh	Mean	2	1.0434	0.3595	1.3588	0.2653	0.0156	0.9845	0.3870	0.6799	0.3829	0.6826	0.3804	0.6844	0.4216	0.6569
	SD	2	0.5392	0.5864	3.1303	0.0514	0.3775	0.6866	1.9646	0.1442	1.7410	0.1794	2.4455	0.0910	2.0120	0.1382
**BackTop-LeftShoulderBack-LeftUpperArmHigh**	**Mean**	2	0.5789	0.5641	0.5947	0.5552	1.8350	0.1649	2.2422	0.1102	2.2731	0.1071	3.1222	**0.0476**	3.2399	**0.0426**
	**SD**	2	2.3301	0.1074	4.5964	**0.0142**	3.9456	**0.0224**	0.4238	0.6554	0.2227	0.8007	0.1632	0.8496	0.3666	0.6939
**BackTop-RightShoulderBack-RightUpperArmHigh**	Mean	2	0.3575	0.7012	0.2573	0.7740	0.6156	0.5424	2.8092	0.0638	2.6764	0.0726	2.9230	0.0576	2.6311	0.0761
	**SD**	2	3.1340	0.0519	6.6653	**0.0025**	0.9057	0.4076	0.2664	0.7666	0.7699	0.4652	2.2086	0.1143	3.1152	0.0479
LeftUpperArmHigh-LeftElbowOut-LeftWristOut	Mean	2	0.6176	0.5432	0.8131	0.4486	0.0917	0.9124	0.2894	0.7492	0.2457	0.7825	0.0682	0.9341	0.0836	0.9199
	SD	2	1.1964	0.3105	1.0386	0.3607	0.6048	0.5481	0.5018	0.6066	0.4446	0.6421	0.6856	0.5057	0.4640	0.6299
RightUpperArmHigh-RightElbowOut-RightWristOut	Mean	2	0.4218	0.6581	0.3834	0.6833	0.1244	0.8831	0.6790	0.5089	0.3996	0.6714	0.0878	0.9160	0.0227	0.9776
	SD	2	0.6450	0.5288	1.2455	0.2956	0.7447	0.4775	0.4103	0.6643	0.0592	0.9425	1.4991	0.2274	1.6201	0.2022

Bold values mean significantly different angles (*P* < 0.05).

**Table 6:** Tukey test results of significantly different mean and SD of each angle in each straightness group (factor: expected emotion).

**Straightness** **group**	**Walking** **direction**	**Type**	**Significant angle** **for 3 markers**	**Significant pair**	***P*-value from** **Tukey test of** **significant pair**
25° to 35°	Counter-clockwise	SD	RightShoulderBack BackTop HeadTop	Negative Video vs Neutral Video	0.0405
					

As shown in Tables 4, 6, in all straightness groups, the difference of *Expected Emotion* only affected the SD of one angle, that is, *RightShoulderBack-BackTop-HeadTop* in the 25° to 35° walking straightness group. In addition, the pair that has a significant effect is *Negative Video vs. Neutral Video*. In other words, only a high curvature walk in the counter-clockwise direction has a different magnitude of head movement between Negative Video and Neutral Video.

**Figure 12:**
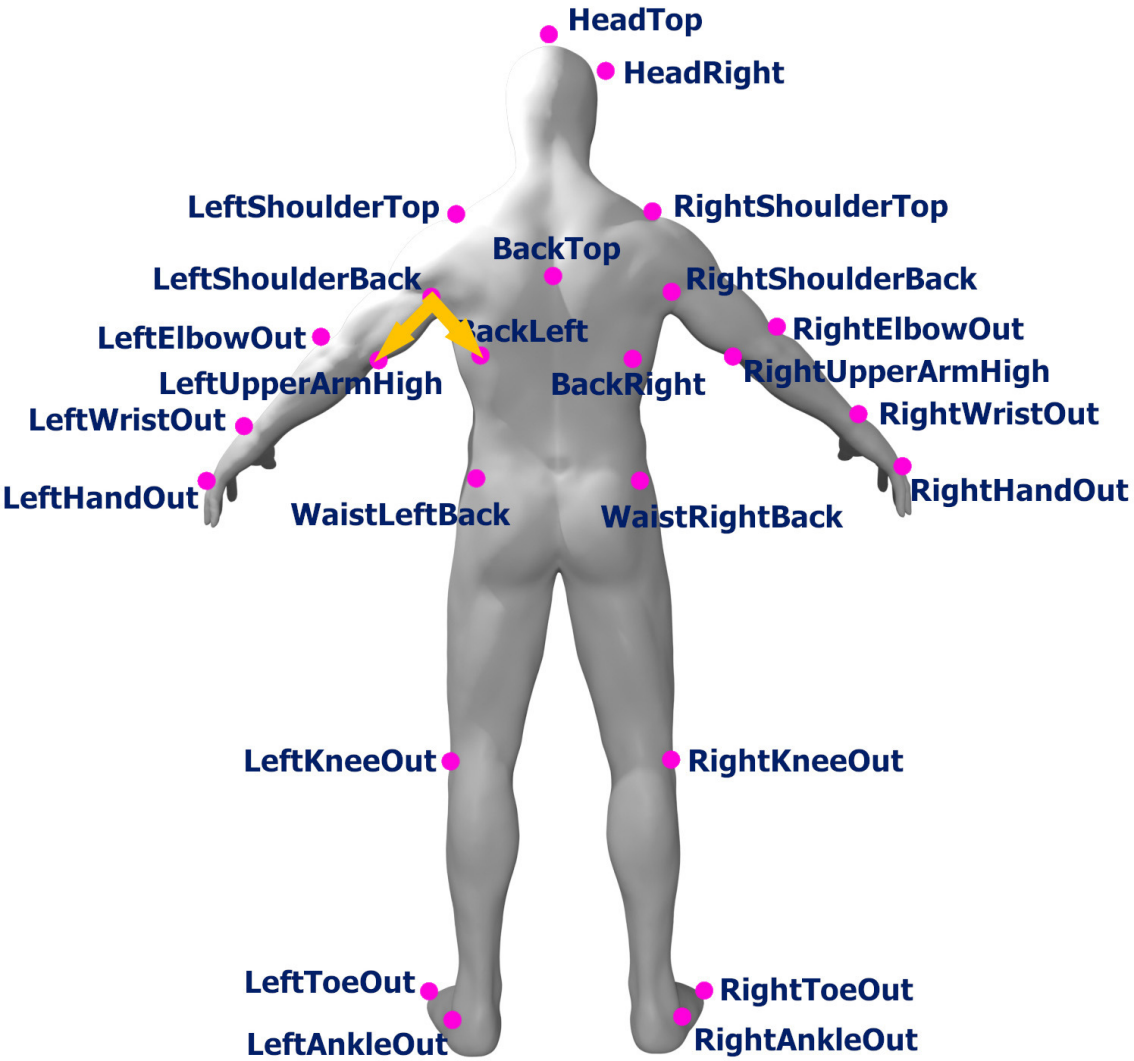
Angle of BackLeft-LeftShoulderBack-LeftUpperArmHigh (arm swing) in body skeleton image (original human figure source: dox012 on Sketchfab^4^).

For *Reported Emotions* analysis, according to Table 5, there are many mean or SD values of angles that are significantly different among the different reported emotions. We also performed the Tukey test with each mean or SD of an angle to check the pairs of emotions that have a significant effect on body movements. Table 7 shows the results from the Tukey test of each mean or SD of an angle. We found that all walking straightness groups have at least one mean or SD of an angle that was affected significantly by happy emotion compared with sad emotion. However, there is only one SD of body part angle that is significantly affected by the emotional differences between *happy and sad* regardless of walking straightness, i.e., the SD of *BackLeft-LeftShoulderBack-LeftUpperArmHigh*, which can be interpreted as *Left Arm Swing Magnitude*. This angle is illustrated in Figure 12.

**Table 7:** Tukey test results of significantly different mean and SD of each angle in each straightness group (factor: reported emotion).

**Straightness group**	**Walking direction**	**Type**	**Significant angle for 3 markers**	**Significant pair**	***P*-value from Tukey test of significant pair**
−35° to −25°	Clockwise	**SD**	**BackLeft** **LeftShoulderBack** **LeftUpperArmHigh**	**Happy vs. Sad**	0.0222
−25° to −15°	Clockwise	Mean	RightAnkleOut RightKneeOut WaistRightFront	Happy vs. Sad Neither vs. Sad	0.0382 0.0070
		SD	WaistLeftFront Chest LeftShoulderTop	Happy vs. Sad	0.0338
		SD	WaistRightFront Chest RightShoulderTop	Happy vs. Sad	0.0095
		SD	LeftKneeOut WaistLeftBack BackLeft	Happy vs. Sad	0.0394
		**SD**	**BackLeft** **LeftShoulderBack** **LeftUpperArmHigh**	**Happy vs. Sad**	0.0017
		SD	BackTop LeftShoulderBack LeftUpperArmHigh	Happy vs. Sad	0.0103
		SD	BackTop RightShoulderBack RightUpperArmHigh	Happy vs. Sad	0.0018
−15° to −5°	Clockwise	**SD**	**BackLeft** **LeftShoulderBack** **LeftUpperArmHigh**	**Happy vs. Sad**	0.0464
		SD	BackTop LeftShoulderBack LeftUpperArmHigh	Happy vs. Neither	0.0313
−5° to 5°	Straight	**SD**	**BackLeft** **LeftShoulderBack** **LeftUpperArmHigh**	**Happy vs. Sad**	0.0258
5° to 15°	Counter-clockwise	**SD**	**BackLeft** **LeftShoulderBack** **LeftUpperArmHigh**	**Happy vs. Sad**	**0.0250**
15° to 25°	Counter-clockwise	Mean	RightShoulderBack BackTop HeadTop	Happy vs. Sad	0.0405
		Mean	BackTop LeftShoulderBack LeftUpperArmHigh	Happy vs. Sad	0.0380
		**SD**	**BackLeft** **LeftShoulderBack** **LeftUpperArmHigh**	**Happy vs. Sad** **Neither vs. Sad**	0.0088 0.0363
25° to 35°	Counter-clockwise	Mean	RightShoulderBack BackTop HeadTop	Happy vs. Sad	0.0442
		Mean	BackTop LeftShoulderBack LeftUpperArmHigh	Happy vs. Sad	0.0342
		**SD**	**BackLeft** **LeftShoulderBack** **LeftUpperArmHigh**	**Happy vs. Sad** **Neither vs. Sad**	0.0027 0.0075

Bold values mean significantly different angles (*P* < 0.05).

**Figure 13:**
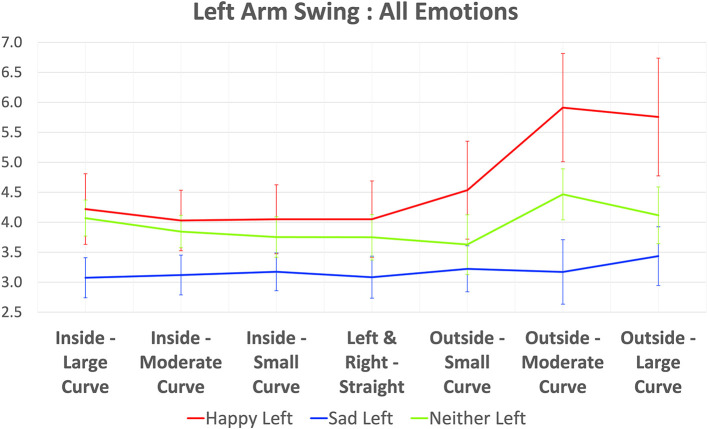
Plot of left arm swing in all emotions (with 95% confident interval error bars).

**Figure 14:**
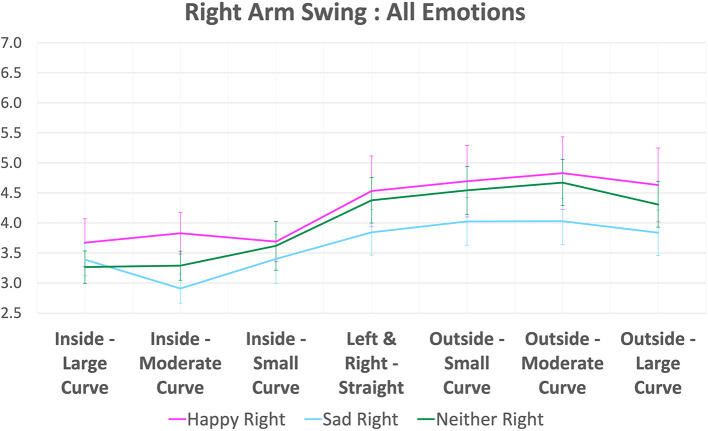
Plot of right arm swing in all emotions (with 95% confident interval error bars).

When subjects walk circularly, one arm's side is inside and the other arm's side is outside of the walking path. We also check the relationship between behavior of left and right arm swings in each emotion with the inside-outside status of that arm and the walking curvatures of the subjects. The inside and outside status of left and right arm can be determined by the walking direction. That is, *the left arm is outside and the right arm is inside* when subjects are walking in the *clockwise* direction, and in the *counter-clockwise* direction, *the left arm is inside and the right arm is outside*. Additionally, the curvature level can be determined from the straightness group. For example, if the angle of straightness is between −35° to −25° or 25° to 35°, it can be considered *large curved walking*, and −5° to 5° can be considered *straight walking*. For the direction, *minus sign means clockwise*, while *plus sign means counter-clockwise*. A list of all curvature levels and walking directions is also shown in Section 5. We plot the arm swing magnitude of the left arm and right arm in all emotions together with its curvature level and its inside-outside status in Figures 13, 14, respectively.

Figure 13 shows the left arm swing magnitude with its inside-outside status and the curvature level. We can see from this figure that when the arm is outside in high curvature walking, the difference between happy and sad is quite obvious. Although the difference between happy and sad is reduced when the curvature decreases, the difference is still large enough to distinguish these emotions. For inside-outside status, we found that when the arm is inside, the difference between happy and sad is smaller than when the arm is outside.

The right arm swing magnitude according to its inside-outside status and the walking curvature are shown in Figure 14. For right arm swing, the differences between happy and sad are smaller than left arm swing at all curvature levels. However, when the subject walks at a high curvature level and the right arm is outside, the arm swing is still higher than when the subject walks in a smaller curvature and the right arm is inside.

### 6.2. Multi-factor analysis of variance (n-way ANOVA)

#### 6.2.1. Methodology

Based on the results of 1-way ANOVA, *Reported Emotion* shows more differences in body part movements between emotions than does *Expected Emotion*. Results of *Expected Emotion* are listed in Table 6, and only one straightness group has a significantly different mean or SD of angle. In Table 7, *Reported Emotion* has one or more significantly different mean or SD of angle in all straightness groups. Therefore, we focused on the analysis of *Reported Emotion* only.

In one-way ANOVA analysis of *Reported Emotion*, some straightness groups had multiple mean or SD values of angle affected by the emotional difference between happy and sad, while other straightness groups had only one mean or SD value affected. The results in Table 7 show that only the left arm swing magnitude *(BackLeft-LeftShoulderBack-LeftUpperArmHigh)* was affected by emotional difference regardless of curvature level and inside-outside status of the arm. In multi-factor ANOVA, we tested several factors including *Reported Emotion, Curvature, Angle Side*, and combinations of these factors to find the relationship between the factors and the left and right arm swing magnitudes. Additionally, we performed multi-factor ANOVA analysis with the arm swing magnitude data of all straightness groups at once.

The factors we used in multi-factor analysis are as follows.

- Reported Emotion: Happy, Sad, Neither
- Curvature: -30, -20, -10, 0, 10, 20, 30
- Angle Side: Left, Right
- Gender: Male, Female
- Reported Emotion × Curvature: Happy/-30, Sad/0, Neither/10, etc.
- Reported Emotion × Angle Side: Happy/Left, Happy/Right, Sad/Left, Sad Right, etc.
- Curvature × Angle Side: -30/Left, -20/Right, 10/Left, etc.
- Reported Emotion × Gender: Happy/Male, Sad/Male, Happy/Female, etc.

First, the *Reported Emotion* factor is for checking whether the arm swing magnitudes are affected by different emotions if we do not consider any other factor.

For the *Curvature* factor, we converted the straightness groups to curvature values before performing multi-factor ANOVA. The sign of curvature value (plus or minus) was selected by checking whether the left arm and right arm were *Inside* or *Outside* when the subjects are walking in a circular walking path. For all curved walking groups, *outside arm used plus sign* and *inside arm used minus sign* as shown in Table 8. For straight walking group, the curvature was 0 for both left arm and right arm, since there was no inside or outside in straight walk. By checking the *curvature* factor, we could verify whether the curvatures of walking, including small curve, moderate curve, and large curve, and the inside-outside status of that arm have significant effects on arm swing magnitude. Since our dataset has a circular walking path that has never been used in conventional studies, we are uncertain whether outside arm swing and inside arm swing during circular walking are symmetric. If the two sides are not symmetric, this implies that one side can be used to more clearly distinguish a subject's current emotion. Moreover, we believe that the curvature of walking can affect arm swinging. For these reasons, we decided to perform an analysis on curvature and the inside-outside status of the arm.

**Table 8:** Conversion from straightness group to curvature for multi-factor ANOVA (plus sign for outside and minus sign for inside).

**Straightness group**	**Actual side**	**Inside/outside**	**Resulting curvature value**
−35° to −25°	Left	Outside	+30
	Right	Inside	-30
−25° to −15°	Left	Outside	+20
	Right	Inside	-20
−15° to −5°	Left	Outside	+10
	Right	Inside	−10
−5° to 5°	Left	-	0
	Right	-	0
5° to 15°	Left	Inside	−10
	Right	Outside	+10
15° to 25°	Left	Inside	−20
	Right	Outside	+20
25° to 35°	Left	Inside	−30
	Right	Outside	+30

For the *Angle Side* factor, because we found from a one-way ANOVA test that only left arm swing is significantly different between happy and sad emotions, we checked whether the arm side (left or right) affects the arm swing magnitude.

Genders of subjects are also possible to have effects with subjects' arm swings while walking, multi-factor ANOVA was also performed with the *Gender* factor to check whether this factor has any significant effect with arm swing magnitudes.

#### 6.2.2. Results

The results from multi-factor ANOVA analysis are listed in Table 9. We found that some factors have a significant effect with arm swing magnitude, including *Reported Emotion, Curvature* and *Gender*. In addition, we found interaction effects between *Reported Emotion with Angle Side, Curvature with Angle Side*, and *Reported Emotion with Gender*.

**Table 9:** Results of multi-factor ANOVA.

**Factor**	**Degrees of freedom**	***F*-value**	***P*-value**
**Reported emotion**	2	42.6435	**0.0000**
**Curvature**	6	8.7467	**0.0000**
Angle side	1	1.9132	0.1668
**Gender**	1	40.9067	**0.0000**
Reported emotion × Curvature	12	0.8662	0.5815
**Reported emotion** **×Angle side**	2	5.7700	**0.0032**
**Curvature** **×Angle side**	6	3.6326	**0.0014**
**Reported emotion** **×Gender**	2	55.7149	**0.0000**

Bold values mean significantly different angles (*P* < 0.05).

We also checked the *Reported Emotion* factor by performing a Tukey test. The results from the Tukey test are shown in Table 10. From this table, every pair of emotion, including Happy vs. Neither, Happy vs. Sad, and Neither vs. Sad, has a significant effect with arm swing magnitude, regardless of any other factors.

**Table 10:** Significantly different pairs from Tukey test of reported emotion.

**Significant pair**	***P*-value from Tukey test**
Happy vs. Neither	0.0011
Happy vs. Sad	0.0000
Neither vs. Sad	0.0000

The *Gender* factor is also checked by Tukey test. Table 11 shows the result of Tukey test for this factor. As shown in this table, male subjects and female subjects have significantly different arm swing magnitudes, regardless of any other factor.

**Table 11:** Significantly different pairs from Tukey test of gender.

**Significant pair**	***P*-value from Tukey test**
Female vs. Male	0.0000

### 6.3. Linear regression analysis

#### 6.3.1. Methodology

Because the behaviors of left arm swings and right arm swings are different, i.e, the left arm has statistically significant arm swing differences among emotions while the right arm does not show such significant differences. We performed linear regression analysis of the left arm and right arm to check whether the regression slopes of each arm side are similar. The regression equation is as follows.


(1) $$\begin{array}{l} Y=\alpha +\beta \times X \end{array}$$

In this equation, α is the intercept, β is the slope, *X* is the curvature value, and *Y* is the predicted arm swing magnitude. That is, we find the most suitable α and β value for each emotion in each arm side that can minimize the differences between predicted arm swing magnitude (*Y*_*Predict*_) and actual arm swing magnitude (*Y*) for each curvature value.

The regression equations for left arm swing magnitude in each emotion are as follows.


(2) $$\begin{array}{l} ArmSwing_{HappyLeft}=\alpha_{HappyLeft}+\beta_{HappyLeft}\times Curvature \end{array}$$

(3) $$\begin{array}{l} ArmSwing_{SadLeft}=\alpha_{SadLeft}+\beta_{SadLeft}\times Curvature \end{array}$$

(4) $$\begin{array}{l} ArmSwing_{NeitherLeft}=\alpha_{NeitherLeft}+\beta_{NeitherLeft}\times Curvature \end{array}$$

The regression equations for right arm swing magnitude in each emotion are as follows.


(5) $$\begin{array}{l} ArmSwing_{HappyRight}=\alpha_{HappyRight}+\beta_{HappyRight}\times Curvature \end{array}$$

(6) $$\begin{array}{l} ArmSwing_{SadRight}=\alpha_{SadRight}+\beta_{SadRight}\times Curvature \end{array}$$

(7) $$\begin{array}{l} ArmSwing_{NeitherRight}=\alpha_{NeitherRight}+\beta_{NeitherRight}\times Curvature \end{array}$$

Accordingly, we find the α and β values of left arm swing and right arm swing for happy, sad, and neither emotion separately. If β_*HappyLeft*_, β_*SadLeft*_, and β_*NeitherLeft*_ are equal, the slopes of all emotions are similar for the left arm. That is, the difference in emotions does not have a significant effect on the left arm swing magnitude in each curvature. For the right arm, we also checked whether β_*HappyRight*_, β_*SadRight*_, and β_*NeitherRight*_ were equal. Consequently, we can follow the same rule used for the left arm to verify whether emotion differences significantly affect the right arm swing.

#### 6.3.2. Results

The results of linear regression of each emotion for the left arm are as follows.

- Happy: α = 4.5521, β = 0.0259
- Neither: α = 3.8916, β = 0.0017
- Sad: α = 3.1764, β = 0.0038.

For the right arm, the linear regression results are as follows.

- Happy: α = 4.2854, β = 0.0206
- Neither: α = 4.0596, β = 0.0227
- Sad: α = 3.6643, β = 0.0142.

According to the above results, for left arm swings, the β values from linear regression of happy and sad are largely different (β = 0.0259 and β = 0.0038, respectively). The results suggest that the left arm swing magnitude when the subjects feel happy is much different from the left arm swing magnitude when the subjects feel sad, since the slope of these two emotions are significantly different. In addition, for right arm swings, the β values of happy and sad are also different, but the difference in the right arm swings' slopes is smaller than that in the left arm swings' slope (β = 0.0206 and β = 0.0142, respectively). Hence, the right arm swing magnitudes under happy and sad emotions are also different from each other, but not so much different as they are in the left arm. Under the neither emotion, the slope of the left arm swings is much different from the slopes of happy and sad emotions (β = 0.0017). However, the slope of the right arm swings is quite similar to the slope of happy emotion (β = 0.0227). Therefore, the neither emotion has significantly different arm swings compared to those of the happy and sad emotions for the left arm side. Nevertheless, for the right arm, the neither emotion has quite similar arm swings to those of the happy emotion.

In summary, the slopes are affected by emotion differences largely for the left arm side, but not so much for the right arm side. These results are in agreement with those from one-way ANOVA.

## 7. Discussion

From the results of all statistical analyses, we examine particularly noteworthy findings as follows.

First, from one-way ANOVA results, we found that human gait is noticeably more affected by the reported emotion, which is from a self-reported questionnaire, than by the expected emotion, which is the annotated emotion of the emotion induction video we used. When we performed a comparison between expected emotions, all subjects watched the same video of each emotion type including neutral, positive and negative video; in each video, the visual patterns, dialogues, sounds, and musical rhythms are identical. Consequently, the results suggest that the gait differences we found from our analyses are not based on the raw video or audio stimuli but on the subjects' reported feelings induced by these videos.

In addition, from one-way ANOVA results of reported emotion, the behaviors of several body part movements are affected by human emotion. These include, for example, the mean values of angles related to the head and shoulders of subjects and the standard deviation values of angles, i.e., movement magnitude related to the chest, waist, and arms of subjects. In particular, the left arm swing magnitude is obviously different among reported emotions regardless of walking curvature. However, only the left arm swing magnitude is significantly affected by the difference in emotions among all curvatures, whereas the right arm swing magnitude is not affected in this way. Since the participants in our study did not walk in a straight walking path but circularly with different curvatures, it is possible that this phenomenon occurred due to subjects' walking curvature.

From these results, we investigated the arm swing magnitude in different walking curvatures. We found from the plots of arm swing magnitude shown in Figures 13, 14 that the inside arm has a smaller arm swing magnitude compared with the outside arm, regardless of reported emotion or the arm side (left or right arm). That is, when comparing within the same emotion, for both left arm and right arm, the arm swing magnitude is smaller if the arm is inside than if the arm is outside. This effect is very obvious for happy emotion. Additionally, arm swing magnitudes of sad emotion are always smaller comparing to happy emotion for both left and right arm according to the experimental results using our dataset. Although the arm swing when the arm is inside is smaller than when the arm is outside in the same emotion, the smallest arm swing of happy emotion remains greater than the largest arm swing of sad emotion as shown in Figures 13, 14. Therefore, we can distinguish the differences between happy and sad emotions from the arm swing magnitudes even we do not know the walking curvature and inside-outside status, but if we have this additional information, it will be easier to distinguish between emotions. Our finding about differences of arm swing magnitudes between happy emotion and sad emotion agrees with previous works in related fields including the studies proposed by Montepare et al. (1987), Michalak et al. (2009) and Halovic and Kroos (2018). All of them have similar findings that subjects' arm swings when feeling sad are smaller than the arm swings when feeling happy. Due to these results of one-way ANOVA, we decided to focus only on arm swing magnitude for reported emotion in the remaining analyses, since we found that arm swing can reveal subjects' emotion better than other body parts. Moreover, the effects of walking curvature and arm side can be investigated from arm swing magnitude using multi-factor ANOVA.

Based on to the results of multi-factor ANOVA, various issues should be considered as follows. First, for the main effects, we found that the reported emotion and curvature have effects on arm swing magnitude. However, the angle side (left or right arm) does not have a significant effect on arm swing magnitude. Furthermore, we found two interaction effects related to the angle side: first, the Reported Emotion factor with Angle Side factor (*F* = 5.3906, *P* = 0.0047); second, the Curvature factor with the Angle Side factor (*F* = 3.4769, *P* = 0.0020). It is possible that these differences are due to the dominant hand of the subjects. Unfortunately, in the current dataset we collected, the dominant hand is not balanced, i.e., most subjects are right-handed. Therefore, this hypothesis cannot be verified and it is still an open question for future investigation. Moreover, the reported emotion and walking curvature do not have interaction effects with each other.

Gender is also another factor that should be investigated. As we want to verify whether female and male subjects have different arm swing magnitudes, multi-factor ANOVA was performed with *Gender* factor in addition to *Reported Emotion, Curvature*, and *Angle Side* factors. From multi-factor ANOVA results, gender is another factor that has significant effect with arm swing magnitudes. Gender factor also has an interaction effect with the reported emotion factor (*F* = 55.7149, *P* = 0.0000). From other related studies, for example, Venture et al. (2014) which used four professional actors including 2 men and 2 women in their experiment, they found that inter-gender recognition is feasible and emotional expressions of male and female subjects are similar. In the study proposed by Gross et al. (2012), most features are not affected by gender e.g., gait velocity, cadence, range of motion at several joints, lateral tilt of pelvis and trunk etc. However, some other features are different among genders such as stride length, elbow range of motion, trunk extension. This study contains 30 subjects with 50% female participants so it should be more reliable as there are more number of subjects. Also, in the study by Kang and Gross (2016) that performed experiment using 11 women and 7 men, some features such as gait speed or stride length are not affected by gender while some other features such as the normal jerk score of elbow and wrist are affected because of gender difference. Hence, it is normal that some features can be affected by gender difference while some features are not. Unfortunately, our study has very imbalanced numbers of male and female subjects so we cannot make the final conclusion about this issue.

As shown in other studies, there are several works about gait analysis which attempted to estimate subjects' ages from their gaits. These studies reveal that human gaits can be used for age estimation (Lu and Tan, 2010; Zhang et al., 2010; Nabila et al., 2018; Gillani et al., 2020). This means that gait patterns from subjects with different age ranges are also different. Because of this issue, emotion recognition accuracy might decrease if the difference of subjects' ages is large. In this study, the average age of participants is 19.69 years with 1.40 years standard deviation as our participants are undergraduate students in our university. It is possible that if the dataset contains more age diversity, the results of investigation could be changed. Therefore, we want to state that the findings from our study are based on a sample group with similar age range.

According to the linear regression analysis, we found that the arm swings in each emotion are affected differently between the left and right arm as the regression slopes of the left arm are very different under each emotion, whereas the regression slopes of the right arm are quite similar among all emotions. This means that the effects of emotions on the left and right arm swings appear differently. The results suggest that we should also consider the arm side in addition to the arm swing magnitude so that we can distinguish between different emotions more easily with higher accuracy.

In summary, according to the results from all analyses, we can confirm the following hypotheses. First, body part movements are different under different emotions. Hence, walking posture can reveal the emotion of subjects. Second, the body part movements of the left and right sides while walking in a non-straight walking path are not symmetric, and thus one side can reveal the emotion of a subject better than the other side.

This study reveals several useful findings for the emotion recognition research field. We found that human gait can reveal the current emotion of subjects while walking, even in a non-straight walking path. Arm swing magnitude shows the differences in subjects' emotions effectively. Consequently, if we know the walking curvature as well as the arm side, it will be easier to distinguish between emotions. The results from our study can be used to develop an emotion recognition system that performs accurately and unobtrusively in real-life situations where the subjects are walking in a crowded environment, without the need for high-quality cameras or specific equipment. Since human emotions can be detected by their arm swing magnitudes, we can use any camera with pose-estimation software to calculate the essential features for emotion prediction. Nevertheless, some issues remain unexplored due to the limitations of our dataset, including the effect of dominant hand and the differences between male and female subjects. We plan to collect more data so we can investigate these issues in the near future.

## 8. Conclusion

In this study, we investigated the differences in body part movements while subjects are walking in a non-straight path and watching emotion-inducing videos using Microsoft HoloLens 2. Since the walking path is not straight, we can collect gait data for different curvatures. Body part movements were captured by the OptiTrack motion-capturing system with 37 markers. For emotion induction using emotion-inducing videos, we found that not all subjects felt the same emotion that we expected them to feel; therefore, it is important to always ask for their feelings after finishing emotion induction. For gait features, we calculated 24 angles that show the movements of body parts while subjects are walking. We also calculated walking straightness and the curvature level of walking, along with the inside-outside status of body side, i.e., left side or right side. According to the results of one-way ANOVA, multi-factor ANOVA, and linear regression analyses on gait data, we found that the magnitudes of arm swing are larger when the subjects are walking and feeling happy than when the subjects are feeling sad. In our opinion, the results agree with human nature that subjects will move slower with less magnitudes when they are feeling sad in comparison to when they are feeling happy. Furthermore, if the subjects walk in a non-straight walking path, observing one side of the body movements will be easier for prediction of emotion than the other side, since the left arm swings can reveal subjects' current emotion better than the right arm swings, especially when the left arm is the outside arm and the subjects are walking at a high curvature level. From all of the analyses we conducted, we conclude that body movements while walking are different under different emotions, so we can detect subjects' emotions using their gait. In particular, arm swing magnitude reveals the current emotions of subjects better than any other part of the body.

## Data availability statement

The raw data supporting the conclusions of this article will be made available by the authors, without undue reservation.

## Ethics statement

The studies involving human participants were reviewed and approved by Future University Hakodate. The patients/participants provided their written informed consent to participate in this study. Written informed consent was obtained from the individual(s) for the publication of any potentially identifiable images or data included in this article.

## Author contributions

NJ conducted the data collection, data preprocessing, feature extraction, and statistical analyses, and wrote this manuscript. KS and AU contributed to the experimental design for data collection, gave suggestions and checked all processes of this study, and reviewed this manuscript before submission. NK and CN made suggestions and checked the data collection, data preprocessing, and feature extraction process, as well as checking this manuscript before submission. All authors contributed to the article and approved the submitted version.

## Funding

This work was supported by JST Moonshot R&D Grant Number JPMJMS2011.

## Conflict of interest

The authors declare that the research was conducted in the absence of any commercial or financial relationships that could be construed as a potential conflict of interest.

## Publisher's note

All claims expressed in this article are solely those of the authors and do not necessarily represent those of their affiliated organizations, or those of the publisher, the editors and the reviewers. Any product that may be evaluated in this article, or claim that may be made by its manufacturer, is not guaranteed or endorsed by the publisher.

## Footnotes

^1^https://sketchfab.com/3d-models/man-5ae6bd9271ac4ee4905b96e5458f435d (last accessed April 6, 2022).

^2^https://www.youtube.com/watch?v=4R9HpESkor8 (last accessed: April 6, 2022).

^3^https://liris-accede.ec-lyon.fr/ (last accessed: April 6, 2022).

^4^https://sketchfab.com/3d-models/man-5ae6bd9271ac4ee4905b96e5458f435d (last accessed: April 6, 2022).

## References

[B1] Ahmed F. Sieu B. Gavrilova M. L. (2018). Score and rank-level fusion for emotion recognition using genetic algorithm, in 2018 IEEE 17th International Conference on Cognitive Informatics &Cognitive Computing (ICCI* CC) (Berkeley, CA: IEEE), 46–53. 10.1109/ICCI-CC.2018.8482086

[B2] Anderez D. O. Kanjo E. Amnwar A. Johnson S. Lucy D. (2021). The rise of technology in crime prevention: opportunities, challenges and practitioners perspectives. arXiv[Preprint].arXiv:2102.04204. 10.48550/arXiv.2102.04204

[B3] Baratin E. Sugavaneswaran L. Umapathy K. Ioana C. Krishnan S. (2015). Wavelet-based characterization of gait signal for neurological abnormalities. Gait Posture 41, 634–639. 10.1016/j.gaitpost.2015.01.012 25661004

[B4] Barliya A. Omlor L. Giese M. A. Berthoz A. Flash T. (2013). Expression of emotion in the kinematics of locomotion. Exp. Brain Res. 225, 159–176. 10.1007/s00221-012-3357-4 23250443

[B5] Baveye Y. Dellandréa E. Chamaret C. Chen L. (2015). Deep learning vs. kernel methods: Performance for emotion prediction in videos, in 2015 International Conference on Affective Computing and Intelligent Interaction (ACII) (Xi'an: IEEE), 77–83. 10.1109/ACII.2015.7344554

[B6] Bouchrika I. (2018). A survey of using biometrics for smart visual surveillance: gait recognition, in Surveillance in Action (Springer), 3–23. 10.1007/978-3-319-68533-5_1

[B7] Bouzakraoui M. S. Sadiq A. Alaoui A. Y. (2019). Appreciation of customer satisfaction through analysis facial expressions and emotions recognition, in 2019 4th World Conference on Complex Systems (WCCS) (Ouarzazate: IEEE), 1–5. 10.1109/ICoCS.2019.8930761

[B8] Busso C. Deng Z. Yildirim S. Bulut M. Lee C. M. Kazemzadeh A.. (2004). Analysis of emotion recognition using facial expressions, speech and multimodal information, in Proceedings of the 6th International Conference on Multimodal Interfaces (State College, PA), 205–211. 10.1145/1027933.1027968

[B9] Chiu M. Shu J. Hui P. (2018). Emotion recognition through gait on mobile devices, in 2018 IEEE International Conference on Pervasive Computing and Communications Workshops (PerCom Workshops) (Athens: IEEE), 800–805. 10.1109/PERCOMW.2018.8480374

[B10] Deluzio K. J. Wyss U. P. Zee B. Costigan P. A. Serbie C. (1997). Principal component models of knee kinematics and kinetics: normal vs. pathological gait patterns. Hum. Movement Sci. 16, 201–217. 10.1016/S0167-9457(96)00051-6

[B11] Destephe M. Maruyama T. Zecca M. Hashimoto K. Takanishi A. (2013). The influences of emotional intensity for happiness and sadness on walking, in 2013 35th Annual International Conference of the IEEE Engineering in Medicine and Biology Society (EMBC) (Osaka: IEEE), 7452–7455. 10.1109/EMBC.2013.6611281 24111468

[B12] Gillani S. I. Azam M. A. Ehatisham-ul Haq M. (2020). Age estimation and gender classification based on human gait analysis, in 2020 International Conference on Emerging Trends in Smart Technologies (ICETST) (Karachi: IEEE), 1–6. 10.1109/ICETST49965.2020.9080735

[B13] Gross M. M. Crane E. A. Fredrickson B. L. (2012). Effort-shape and kinematic assessment of bodily expression of emotion during gait. Hum. Movement Sci. 31, 202–221. 10.1016/j.humov.2011.05.001 21835480

[B14] Halovic S. Kroos C. (2018). Not all is noticed: kinematic cues of emotion-specific gait. Hum. Movement Sci. 57, 478–488. 10.1016/j.humov.2017.11.008 29174557

[B15] Isaac E. R. Elias S. Rajagopalan S. Easwarakumar K. (2019). Multiview gait-based gender classification through pose-based voting. Pattern Recogn. Lett. 126, 41–50. 10.1016/j.patrec.2018.04.020

[B16] Ismail A. R. Asfour S. S. (1999). Discrete wavelet transform: a tool in smoothing kinematic data. J. Biomech. 32, 317–321. 10.1016/S0021-9290(98)00171-7 10093032

[B17] Janssen D. Schöllhorn W. I. Lubienetzki J. Fölling K. Kokenge H. Davids K. (2008). Recognition of emotions in gait patterns by means of artificial neural nets. J. Nonverbal Behav. 32, 79–92. 10.1007/s10919-007-0045-3

[B18] Kang G. E. Gross M. M. (2015). Emotional influences on sit-to-walk in healthy young adults. Hum. Movement Sci. 40, 341–351. 10.1016/j.humov.2015.01.009 25681657

[B19] Kang G. E. Gross M. M. (2016). The effect of emotion on movement smoothness during gait in healthy young adults. J. Biomech. 49, 4022–4027. 10.1016/j.jbiomech.2016.10.044 27823805

[B20] Karg M. Kühnlenz K. Buss M. (2010). Recognition of affect based on gait patterns. IEEE Trans. Syst. Man Cybernet. B 40, 1050–1061. 10.1109/TSMCB.2010.2044040 20350859

[B21] Khamsemanan N. Nattee C. Jianwattanapaisarn N. (2017). Human identification from freestyle walks using posture-based gait feature. IEEE Trans. Inform. Forensics Sec. 13, 119–128. 10.1109/TIFS.2017.2738611

[B22] Kim S. Nussbaum M. A. Ulman S. (2018). Impacts of using a head-worn display on gait performance during level walking and obstacle crossing. J. Electromyogr. Kinesiol. 39, 142–148. 10.1016/j.jelekin.2018.02.007 29501988

[B23] Kitchat K. Khamsemanan N. Nattee C. (2019). Gender classification from gait silhouette using observation angle-based geis, in 2019 IEEE International Conference on Cybernetics and Intelligent Systems (CIS) and IEEE Conference on Robotics, Automation and Mechatronics (RAM) (Bangkok: IEEE), 485–490. 10.1109/CIS-RAM47153.2019.9095797

[B24] Kuijsters A. Redi J. De Ruyter B. Heynderickx I. (2016). Inducing sadness and anxiousness through visual media: measurement techniques and persistence. Front. Psychol. 7, 1141. 10.3389/fpsyg.2016.01141 27536260 PMC4971078

[B25] Lemke M. R. Wendorff T. Mieth B. Buhl K. Linnemann M. (2000). Spatiotemporal gait patterns during over ground locomotion in major depression compared with healthy controls. J. Psychiatr. Res. 34, 277–283. 10.1016/S0022-3956(00)00017-0 11104839

[B26] Li B. Zhu C. Li S. Zhu T. (2016). Identifying emotions from non-contact gaits information based on microsoft kinects. IEEE Trans. Affect. Comput. 9, 585–591. 10.1109/TAFFC.2016.2637343

[B27] Li S. Cui L. Zhu C. Li B. Zhao N. Zhu T. (2016). Emotion recognition using kinect motion capture data of human gaits. PeerJ 4, e2364. 10.7717/peerj.2364 27672492 PMC5028730

[B28] Limcharoen P. Khamsemanan N. Nattee C. (2020). View-independent gait recognition using joint replacement coordinates (JRCs) and convolutional neural network. IEEE Trans. Inform. Forensics Sec. 15, 3430–3442. 10.1109/TIFS.2020.2985535

[B29] Limcharoen P. Khamsemanan N. Nattee C. (2021). Gait recognition and re-identification based on regional lstm for 2-second walks. IEEE Access 9, 112057–112068. 10.1109/ACCESS.2021.3102936

[B30] Lu J. Tan Y.-P. (2010). Gait-based human age estimation. IEEE Trans. Inform. Forensics Sec. 5, 761–770. 10.1109/TIFS.2010.2069560

[B31] Michalak J. Troje N. F. Fischer J. Vollmar P. Heidenreich T. Schulte D. (2009). Embodiment of sadness and depression–gait patterns associated with dysphoric mood. Psychosom. Med. 71, 580–587. 10.1097/PSY.0b013e3181a2515c 19414617

[B32] Montepare J. M. Goldstein S. B. Clausen A. (1987). The identification of emotions from gait information. J. Nonverbal Behav. 11, 33–42. 10.1007/BF00999605

[B33] Nabila M. Mohammed A. I. Yousra B. J. (2018). Gait-based human age classification using a silhouette model. IET Biometrics 7, 116–124. 10.1049/iet-bmt.2016.0176

[B34] Nyan M. Tay F. Seah K. Sitoh Y. (2006). Classification of gait patterns in the time-frequency domain. J. Biomech. 39, 2647–2656. 10.1016/j.jbiomech.2005.08.014 16212968

[B35] Olney S. J. Griffin M. P. McBride I. D. (1998). Multivariate examination of data from gait analysis of persons with stroke. Phys. Therapy 78, 814–828. 10.1093/ptj/78.8.814 9711207

[B36] Picard R. W. (2000). Affective Computing. MIT Press. 10.7551/mitpress/1140.001.0001

[B37] Quiroz J. C. Geangu E. Yong M. H. (2018). Emotion recognition using smart watch sensor data: mixed-design study. JMIR Mental Health 5, e10153. 10.2196/10153 30089610 PMC6105867

[B38] Roether C. L. Omlor L. Christensen A. Giese M. A. (2009). Critical features for the perception of emotion from gait. J. Vision 9, 15–15. 10.1167/9.6.15 19761306

[B39] Sadeghi H. Allard P. Duhaime M. (1997). Functional gait asymmetry in able-bodied subjects. Hum. Movement Sci. 16, 243–258. 10.1016/S0167-9457(96)00054-1

[B40] Sedighi A. Rashedi E. Nussbaum M. A. (2020). A head-worn display (“smart glasses”) has adverse impacts on the dynamics of lateral position control during gait. Gait Posture 81, 126–130. 10.1016/j.gaitpost.2020.07.014 32717669

[B41] Sedighi A. Ulman S. M. Nussbaum M. A. (2018). Information presentation through a head-worn display (“smart glasses”) has a smaller influence on the temporal structure of gait variability during dual-task gait compared to handheld displays (paper-based system and smartphone). PLoS ONE 13, e0195106. 10.1371/journal.pone.0195106 29630614 PMC5891005

[B42] Shiavi R. Griffin P. (1981). Representing and clustering electromyographic gait patterns with multivariate techniques. Med. Biol. Eng. Comput. 19, 605–611. 10.1007/BF02442775 7334869

[B43] Stephens-Fripp B. Naghdy F. Stirling D. Naghdy G. (2017). Automatic affect perception based on body gait and posture: a survey. Int. J. Soc. Robot. 9, 617–641. 10.1007/s12369-017-0427-6

[B44] Sun B. Zhang Z. Liu X. Hu B. Zhu T. (2017). Self-esteem recognition based on gait pattern using kinect. Gait Post. 58, 428–432. 10.1016/j.gaitpost.2017.09.001 28910655

[B45] Tiam-Lee T. J. Sumi K. (2019). Analysis and prediction of student emotions while doing programming exercises, in International Conference on Intelligent Tutoring Systems (Kingston: Springer), 24–33. 10.1007/978-3-030-22244-4_4

[B46] Venture G. Kadone H. Zhang T. Grézes J. Berthoz A. Hicheur H. (2014). Recognizing emotions conveyed by human gait. Int. J. Soc. Robot. 6, 621–632. 10.1007/s12369-014-0243-1

[B47] Wootten M. Kadaba M. Cochran G. (1990). Dynamic electromyography. I. Numerical representation using principal component analysis. J. Orthopaed. Res. 8, 247–258. 10.1002/jor.1100080214 2303958

[B48] Xu S. Fang J. Hu X. Ngai E. Guo Y. Leung V.. (2020). Emotion recognition from gait analyses: current research and future directions. arXiv[Preprint].arXiv:2003.11461. 10.48550/arXiv.2003.11461

[B49] Yelwande S. S. Dandavate R. M. (2020). Study of emotion recognition models for socially aware robots and subsequent path mapping, in 2020 4th International Conference on Electronics, Communication and Aerospace Technology (ICECA) (Coimbatore: IEEE), 1230–1233. 10.1109/ICECA49313.2020.9297630

[B50] Zhang D. Wang Y. Bhanu B. (2010). Age classification base on gait using HMM, in 2010 20th International Conference on Pattern Recognition (Istanbul: IEEE), 3834–3837. 10.1109/ICPR.2010.934

[B51] Zhang Z. Song Y. Cui L. Liu X. Zhu T. (2016). Emotion recognition based on customized smart bracelet with built-in accelerometer. PeerJ 4, e2258. 10.7717/peerj.2258 27547564 PMC4974923

